# Parallel residual projection: a new paradigm for solving linear inverse problems

**DOI:** 10.1038/s41598-020-69640-5

**Published:** 2020-07-30

**Authors:** Wei Miao, Vignesh Narayanan, Jr-Shin Li

**Affiliations:** Department of Electrical and Systems Engineering, Washington University in St. Louis, St. Louis, MO 63130 USA

**Keywords:** Applied mathematics, Computational science, Network topology

## Abstract

A grand challenge to solve a large-scale linear inverse problem (LIP) is to retain computational efficiency and accuracy regardless of the growth of the problem size. Despite the plenitude of methods available for solving LIPs, various challenges have emerged in recent times due to the sheer volume of data, inadequate computational resources to handle an oversized problem, security and privacy concerns, and the interest in the associated incremental or decremental problems. Removing these barriers requires a holistic upgrade of the existing methods to be computationally efficient, tractable, and equipped with scalable features. We, therefore, develop the parallel residual projection (PRP), a parallel computational framework involving the decomposition of a large-scale LIP into sub-problems of low complexity and the fusion of the sub-problem solutions to form the solution to the original LIP. We analyze the convergence properties of the PRP and accentuate its benefits through its application to complex problems of network inference and gravimetric survey. We show that any existing algorithm for solving an LIP can be integrated into the PRP framework and used to solve the sub-problems while handling the prevailing challenges.

## Background

A wide variety of commonly encountered model-based or data-driven problems in scientific computing, across diverse fields, such as recovering the dynamic topology of complex networks from measured data^1,2^, image reconstruction^3^, data-driven discovery of ordinary^4,5^ and partial differential equations^4–6^, seismic tomography^7^, and gravimetric survey^8,9^, can ultimately be posed as a linear inverse problem (LIP). Accentuated by the myriad application domains, methods to solve the LIP have been extensively developed over the past several decades. A menu of algorithms, which can be broadly classified into direct and iterative methods, is available. Representative direct methods include the algorithms invented based on linear algebra, such as the Gaussian elimination, LU factorization^10^, and the truncated singular value decomposition^11^, or sparse optimization, such as the renowned compressed sensing^12–14^, for directly calculating the solution at once. On the other hand, iterative methods such as the Kaczmarz^15^, coordinate descent^16^, iterative shrinkage-thresholding (IST)^17,18^, and conjugate gradient-based^19,20^ algorithms, are tailored to improve computational efficiency by using recursive update rules to reach a convergent solution.

With the advent of *big data*, there has been a growing interest in developing algorithms that can be implemented on a distributed computational platform^21^. In many emerging applications, the abundance of data generated during scientific experiments leads to an exaggeratedly oversized LIP. For example, in the problem of inferring the dynamic topology of a complex network^2^ composed of 5,000 nodes given a time-series data of 1,000,000 sampling points (observations), the regression matrix *A* (corresponding to one of the 5,000 associated LIPs) alone will occupy $5{,}000\times 1{,}000{,}000\times 8 ~ \text {bytes} (40~\text {GB})$ storage space (if stored in double-precision format), let alone the discussions of computational issues. To handle such large volumes of data, distributed computational methods were extensively proposed, allowing the implementation of aforementioned algorithms on a distributed platform for parallel computation^21–23,25^.

Although one may claim victory on these developments, most of the existing computational methods were invented to tackle the LIP with a well-defined dimension, but not specifically tailored to be adaptive and scalable towards solving the associated incremental or decremental problems (see Supplementary Information, Section S.1). Also, besides storage management, the computational resources required to accommodate a giant problem are far beyond the capability of ubiquitous low-end computing devices. For example, an exploratory rover on an extraterrestrial surface may require solving a large-scale LIP associated with seismic tomography or gravimetric survey while navigating in an uncertain environment with a limited computing memory bank. Therefore, an efficient computational resources utilization strategy is desired to solve the LIP in resource-constrained applications.

The conventional practice to remove the impediment due to limited computing resources is to employ a cluster of high-end servers. Distributed algorithms such as consensus-based optimization^22,23,26^, and distributed iterative algorithms^21^ were developed in an attempt to handle oversized linear systems of equations on distributed computing platforms. However, in addition to the tremendous cost, complete information of either the regression matrix or the solution vector is required to be passed to an external device (cloud-server or a cluster of servers), which may potentially trigger security concerns and privacy issues^27,28^, especially in applications involving sensitive information, such as financial records for business, individually identifiable health information, or in defense sectors.

**Figure 1:**
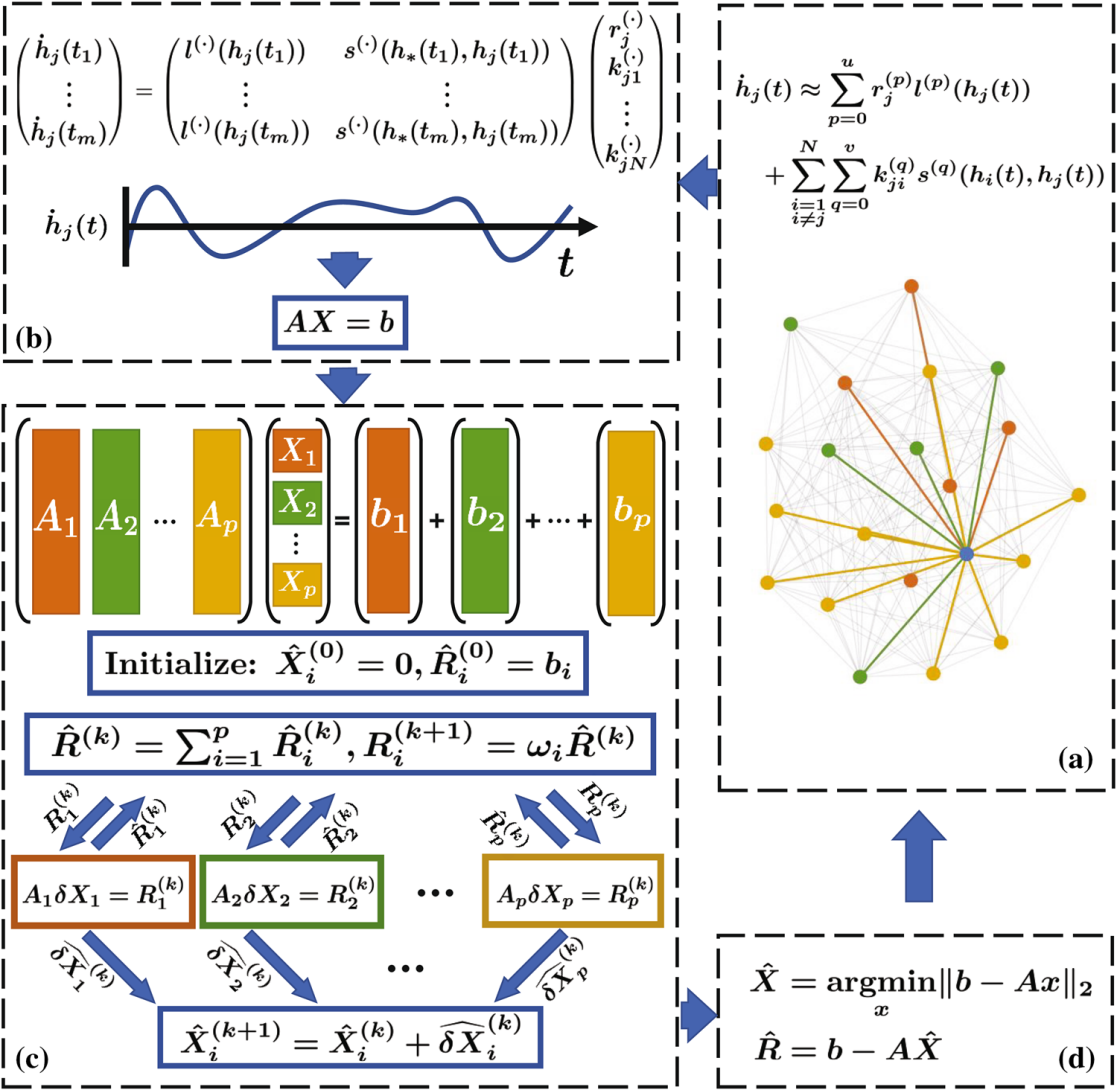
The PRP algorithm decomposes solving the linear inverse problem into *p* linear inverse sub-problems, solutions of which can be then fused to obtain the least squares solution of $Ax = b$. For instance, in a network inference problem shown in (**a**), inferring the connections from a specific node (shown as the blue node) based on its time series observations $h_j(t)$ can be formulated as an inverse problem $Ax = b$ as demonstrated in (**b**). Here $l^{(\cdot )}, s^{(\cdot )}$ correspond to the columns of *A*, $r_j^{(\cdot )}, k_{ji}^{(\cdot )}$ correspond to the unknowns *x*, and ${\dot{h}}_j(t)$ correspond to the observations *b*. Then as illustrated in (**c**), sub-problems are dispatched to several agents, where each agent infers connections between the target node and a cluster of the remaining nodes [shown as red, green and yellow nodes in (**a**)]. In this sense, the regression matrix *A* is partitioned into column blocks $A_1, \ldots , A_p$ that are distributedly stored in *p* computing agents. These *p* agents solve *p* sub-problems taking the form $A_i\delta X_i = R_i^{(k)}$ in parallel, where $R_i^{(0)}$ is initialized by decomposing observations *b* into $b_1, \ldots , b_p$ satisfying $b = \sum _{i=1}^p b_i$. By updating the intermediate solutions and the residuals ${\hat{X}}_i^{(k)}, {\hat{R}}_i^{(k)}$ with a centralized computing device, and re-initializing the *p* sub-problems, the least squares solution and the minimum residual, ${\hat{X}}$ and ${\hat{R}}$, of $Ax = b$ are acquired in (**d**). The solution ${\hat{X}}$ indicates the estimation of coupling strengths between the blue node and the other nodes, which is accessible only to one computing device.

Apart from voluminous data and limited computational resources, another frequently experienced and essential challenge arising from many applications that involve solving an LIP is the variability in the size of the problem. This happens when, for example, additional unknown variables are introduced or existing ones are removed. These “local” changes lead to an incremental or a decremental problem with the size of the original problem expanded or reduced, respectively. Specifically, consider the LIP,

1 $$\begin{aligned} Ax=b, \end{aligned}$$

where $A\in {\mathbb {R}}^{m\times n}$ is the regression map, $b \in {\mathbb {R}}^m$ is the vector of observations, and $x \in {\mathbb {R}}^n$ is the vector of unknown variables (unknown coordinates). In this problem, the addition or deletion of unknown variables leads to a change in the number of columns (i.e., *n*) of *A*; analogously, the inclusion of additional observations as more data become available or deletion of outliers due to measurement error/noise increases or decreases the number of rows (i.e., *m*) of *A*, respectively. For instance, in the experiments involving a gravimetric survey to study the density of the underground geology and surrounding terrain^8, 9^, adding/removing hypothetical subterranean mass is frequently encountered, which corresponds to adding/deleting columns of *A* in the associated LIP. In the context of network inference^2^, the size of the matrix *A* in the LIP (1) is determined by the available observation data points *m* and the number of nodes *N* through the relation $n=(u+1) + (v+1)(N-1)$, where *u*, *v* are the numbers of the spectral approximation terms representing the network dynamics (see Fig. 1). As a result, when a single node is added to or removed from the network, the updated LIP, with $v+1$ columns added or removed, has to be re-solved.

In this context, solving a large-scale problem modeled as an LIP in (1) is admittedly cumbersome, which may cost hours, even days, in many cases. On top of this, the challenges arising from sheer volume of data, inadequate computational resources, security and privacy concerns, and the variability in the size of the LIP inspire a compelling need for developing a systematic and adaptive strategy to not only solve massive LIPs but also update the solution to the related incremental or decremental problems to avoid gratuitous computational time and cost.

One fundamental approach to tackle a problem of huge size is to decompose the problem into smaller, easily tractable sub-problems, and then develop a strategy that utilizes the solutions of the sub-problems to arrive at the solution or an approximate solution of the original problem. Such an approach involving decomposition and fusion is the inception of the distributed optimization algorithms^21–25,29^. However, these algorithms were not catered to address issues related to security and privacy concerns, and not equipped with the capability to solve for incremental or decremental problems through adaptive updates of the current solution in a computationally efficient manner.

In this work, inspired by the randomized coordinate descent and the randomized Kaczmarz algorithms^30,31^, we propose a universal computational framework compatible with any existing method, called the *Parallel Residual Projection (PRP)*, which enables a distributed, parallel computation of the solution to an exaggeratedly oversized LIP. Specifically, the PRP decomposes a given LIP into multiple linear inverse sub-problems of much smaller size and complexity. These sub-problems are then solved independently in multiple stages, in a decentralized manner. The solutions of these sub-problems are then fused by the proposed update strategy so as to converge to the solution of the original problem. Such decomposition into sub-problems breaks through the limitation of computational resources and, moreover, provides a tractable procedure that returns a solution at an arbitrary accuracy. Most importantly, equipped with an appropriate iterative method for solving the sub-problems, the PRP can be contrived to tackle the incremental or decremental problems in a computationally efficient manner, and can be shown to be a powerful, adaptive, and scalable method.

## Methods

The PRP algorithm begins by partitioning the regression matrix *A* into column blocks such that $A = [A_1, \ldots , A_p]$, where $A_i \in {\mathbb {R}}^{m \times d_i}$ with $\sum _{i=1}^p d_i = n$. Each of these partitioned matrices can be considered as the data stored in *p* computing agents (see Fig. 1). The solution vector *x* is in turn decomposed into ${\hat{X}}_1, \ldots , {\hat{X}}_p$ where ${\hat{X}}_i \in {\mathbb {R}}^{d_i}$, and the observation vector *b* is represented as $b=\sum _{i=1}^p b_i$, where $b_i\in {\mathbb {R}}^m$. Initializing with $R_i^{(0)}=b_i$ for $i=1,\ldots ,p$, the *p* intermediate sub-problems of the form $A_i\delta X_i^{(k)}=R_i^{(k)}$ are solved in parallel by *p* agents, where *k* denotes the stage number and $\delta X_i^{(k)}$ denotes the vector of unknown variables of the $i{\mathrm{th}}$ sub-problem. We denote the solution of the $i{\text {th}}$ sub-problem along with the associated residual as the pair $(\widehat{\delta X_i}^{(k)},{\hat{R}}_i^{(k)})$, and the total residual after the $k{\mathrm{th}}$ stage as ${\hat{R}}^{(k)} = \sum _{i=1}^p {\hat{R}}_i^{(k)}$. Then, the update rule for the solution and the residual to the LIP in (1), at the $(k+1){\mathrm{th}}$ stage, is given by

2 $$\begin{aligned} {\hat{X}}_i^{(k+1)} = {\hat{X}}_i^{(k)} + \widehat{\delta X_i}^{(k)}, \quad R_i^{(k+1)} = w_i{\hat{R}}^{(k)}, ~~~i = 1,\ldots , p, \end{aligned}$$

with ${\hat{X}}_i^{(0)} = 0$, where $w_i\in (0,1)$, $i = 1, \ldots , p$ and $\sum _{i=1}^p w_i = 1$.

Geometrically speaking, the PRP can be understood as an iterative projection of the residual components on the column-space of the regression matrix in multiple stages. Specifically, at each stage, the residual is divided and distributed to *p* agents, and these residual parts are projected onto the corresponding column-subspaces of the matrix *A* simultaneously. At the end of each stage, the residuals are collected and used for the reinitialization of the next stage using the update rule in (2). Following this procedure, the individual residual corresponding to each sub-problem that is not orthogonal to the subspaces of other sub-problems, is carried forward to the next stage, and is projected onto each column-subspace in the subsequent stages. As a result, the total residual ${\hat{R}}^{(k)}$ asymptotically becomes orthogonal to all columns of the regression matrix as *k* goes to infinity, which corresponds to the minimal residual of the LIP and leads to the least-squares solution for the LIP in (1) (see Supplementary Information, Section S.2).

In practice, such a decomposition procedure enables an integration of all available computational resources at the user’s disposal into the PRP framework for solving the LIP in (1). For instance, to solve an LIP when *m* is 1,000,000 and *n* is 5,000 (occupying 40 GB in MATLAB) by utilizing a few desktops, say 10, with 8 GB memory each, the PRP distributes this problem in such a way that each of the computing devices is required to handle a feasible sub-problem of the size 1,000,000 by 500 (taking 4 GB memory). Similarly, when multiple cloud-servers are available, each server needs to have access to only one column block of the regression matrix, and have access only to the intermediate solution at the end of each stage. As a result, neither the complete input information, say *A*, *b*, nor the final solution, say *x*, is available to any of the cloud-servers, offsetting any security and privacy concerns.

In addition to the advantages mentioned above, a key attribute of the PRP, coming from its simple geometric interpretation, is that the algorithm converges regardless of how the matrix *A* is partitioned and which method is adopted to solve the *p* sub-problems (see Supplementary Information, Section S.2, Theorem S.1). Therefore, even if the decomposition results in a sub-problem with a large matrix that cannot be handled by a single computing agent, existing parallel computing methods, such as the distributed Kaczmarz algorithms^7^, can be employed to further break-down the sub-problems. Alternatively, when applicable, any of the direct methods can be used to compute the least-squares solution for the sub-problems. Notably, it can be established that the intermediate solution ${\hat{X}}^{(k)}$ and the residual ${\hat{R}}^{(k)}$ converge to the least-squares solution and the minimum residual exponentially with respect to the stage number *k*, respectively. Furthermore, the number of stages required to achieve the desired accuracy can be computed irrespective of the methods used to solve the sub-problems (see Supplementary Information, Section S.2, Theorem S.2).

A particular iterative method for solving the sub-problems is the randomized residual projection (RRP) algorithm (see Supplementary Information, Section S.3). Using the RRP algorithm within the PRP framework, we are able to not only quantify the relationship between ‘the initial residual’ and ‘the number of iterations required for achieving a prescribed accuracy’ (see Supplementary Information, Section S.3, Theorems S.3, S.4, and S.5), but also provide an explicit, adaptive update rule toward finding solutions to the incremental or decremental problems. In this process, we also develop the parallelized RRP (PRRP), which was not addressed in the literature (see Supplementary Information, Section S.5). Most importantly, the utilization of the PRRP enables the PRP to be a ‘scalable’ method, by which the solutions to the related incremental or decremental problems can be found within a guaranteed number of iterations, which requires much less computational time and complexity compared to re-solving them (see Supplementary Information, Sections S.4.1 and S.4.2). In particular, when RRP is parallelized by the PRP framework, if the partitions of *A* satisfy the condition,

3 $$\begin{aligned} \max _{i\in \{1,\ldots ,p\}} \big \Vert w_iA_i(A_i'A_i)^{-1}A_i' {\hat{R}}^{(k)}\big \Vert _2 \le \big \Vert \sum _{i=1}^p w_iA_i (A_i'A_i)^{-1}A_i' {\hat{R}}^{(k)}\big \Vert _2, \end{aligned}$$

the computational time required to complete one stage of the PRP (i.e., to reduce the total residual ${\hat{R}}^{(k)}$ to ${\hat{R}}^{(k+1)}$) is less than the time needed when the RRP is directly applied (see Supplementary Information, Section S.5, Theorem S.6).

## Results

In this section, we present several numerical experiments to demonstrate the flexibility of the proposed PRP framework to solve large-scale LIPs and its application to network topology inference and gravimetric survey. All the numerical experiments were implemented on a single workstation with Xeon Gold 6144 (24.75M Cache, 3.5 GHz), 192 GB memory.

Example 1: Performance analysis of PRP We first present simulation results on the running time of the PRP algorithm. In particular, we generated *A* as a random matrix of dimension $100{,}000 \times 50{,}000$ ($\approx 40$ GB memory storage in MATLAB). Each element of *A* was selected as a random number under a uniform distribution on [0, 1]. A sparse vector *z* of dimension $50{,}000\times 1$ was then generated which consisted of $99\%$ of elements as 0’s, and 1’s as the remaining elements. The vector of observations *b* was generated by $b = Az + \omega$, where $\omega$ was set as a $100{,}000\times 1$ random vector with elements uniformly distributed in [0, 0.2].

**Figure 2:**
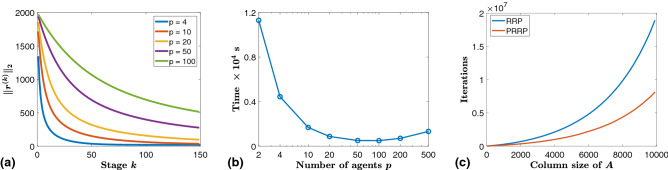
A random matrix *A* of dimension $100{,}000 \times 50{,}000$ was generated ($\approx 40$ GB memory storage in MATLAB), where each element of *A* was selected as a random number under a uniform distribution on [0, 1]. A sparse vector *z* of dimension $50{,}000\times 1$ was then generated in which $99\%$ of the elements were set as 0, and the remaining elements were set as 1. The vector of observations, denoted as *b*, was defined as $b = Az + \omega$, where $\omega$ was chosen as a $100{,}000\times 1$ random vector with elements uniformly distributed in [0, 0.2]. The PRP algorithm, where sub-problems are solved by directly computing the pseudo-inverse of $A_i$, was applied to solve the linear inverse problem $Ax = b$. (**a**) Residuals at different stages (of the PRP algorithm) when various numbers of agents were used to solve the sub-problems, where the matrix *A* of size $100{,}000 \times 50{,}000$ was partitioned into blocks of *p* columns of size $100{,}000\times 50{,}000/p$ in each case. (**b**) Convergence time (in s) when the total residual becomes smaller than $0.5\%$ of the initial residual using the PRP algorithm with various number of partitions *p*. (**c**) Number of iterations required to achieve a residual error $\epsilon < 1\times 10^{-4}$ when using the RRP and the PRRP (with $p = 2$) algorithms to solve the LIPs with different sizes of the matirx *A*.

We used the PRP algorithm to solve this LIP and recorded the convergence behavior of the residuals for various partition sizes. The main results are displayed in Fig. 2 (see also Supplementary Information, Section S.6.1). Figure 2a depicts the convergence of the residual with respect to the number of stages *k*. Figure 2b displays the cumulative time to shrink the total residual to a desired threshold for various number of partitions *p*, which indicates that finer partitions do not necessarily lead to faster convergence of the algorithm. This is because, in the case where finer partitions are defined, although the original LIP is divided into smaller sub-problems that can be solved in less time, it requires more stages in order to reduce the total residual to within a desired threshold, as evidenced in Fig. 2a. To see how the parallel framework of the PRP accelerates the solving of a large-scale LIP, we provide a comparison between the RRP algorithm and the PRRP algorithm with $p=2$ in Fig. 2c. We specifically varied the size of the matrix *A* and solved the resulting LIPs using the RRP algorithm and the proposed PRRP algorithm. For each of the LIPs, we recorded the number of iterations required to bring the residual error within a threshold defined by $\epsilon = 1\times 10^{-4}$ using the RRP algorithm (see Algorithm S.2 in Supplemental Material). Similarly, for the PRRP algorithm, we recorded the cumulative number of iterations required to achieve a total residual error satisfying $\Vert {\hat{R}}\Vert _2 < 1\times 10^{-4}$, where this number was computed by adding the number of iterations in each of the sub-problems. We observed from Fig. 2c that due to the parallelization of computations, the PRRP algorithm required around $50\%$ fewer iterations to reach the desired accuracy compared with the use of the RRP algorithm.

Example 2: Network inference To accentuate the effectiveness of the PRP framework, we present an emerging application in network inference^1, 2^. We consider a complex network constituted by a large number of structurally identical dynamical units that interact with each other. An essential objective of the network inference problem is to find out time-varying interactions between units, and to identify the significant connections/couplings to recover the network topology, using the time-series data of each node in the network (see Fig. 1). For a network with *N* nodes, the inference can be encoded into *N* LIPs^2^. Since the ultimate goal is to decode the coupling topology, it is imperative to not only effectively solve the corresponding large-scale LIP, but also have the flexibility to track the accuracy of the solution and terminate the algorithm when a desired accuracy is met. Such an early termination helps recover network topology swiftly, in particular, in terms of identifying dominating nodes that govern the network dynamics (see Supplementary Information, Section S.6.2). It is also a key feature of the PRP that new estimates of the network topology can be adaptively updated based on the current estimate when more observations become available or when the network is expanded or reduced due to addition or deletion of nodes, respectively.

In our numerical experiment, we generated a synthetic network of Kuramoto oscillators^33^. In particular, we defined a small-world network based on Watts–Strogatz model^32^, which was used to characterize the coupling topology of the Kuramoto oscillator network. The total number of nodes was fixed as 300. We considered two cases corresponding to a dense and a sparse network. We then generated time-series data with 600,000 data points from each of the 300 nodes. The problem of inferring the topology of this network was then mapped into 300 LIPs^2^, each with a regression matrix of size $600{,}000\times 300$. The detailed parameterization of the oscillator network and the formulation of this network inference problem into LIPs are provided in the Supplementary Information, Section S.6.2.

**Figure 3:**
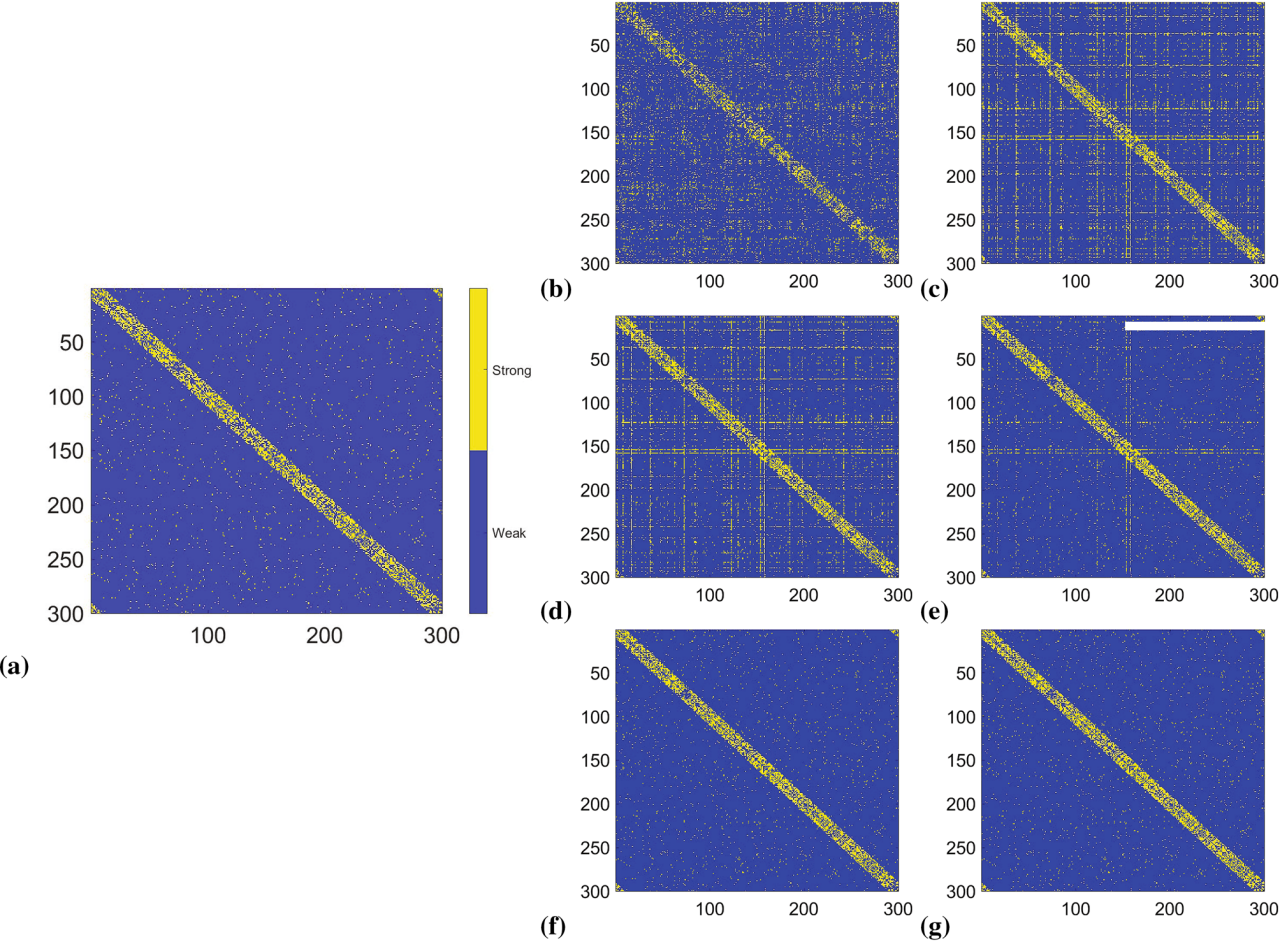
(**a**) Original topology of dense Kuramoto oscillator network indicating if the connections are either strong or weak. Each point indicates whether two nodes are strongly or weakly coupled. (**b**–**g**) Network topology recovered using the PRP algorithm after 10, 50, 100, 200, 300, 450 stages.

**Figure 4:**
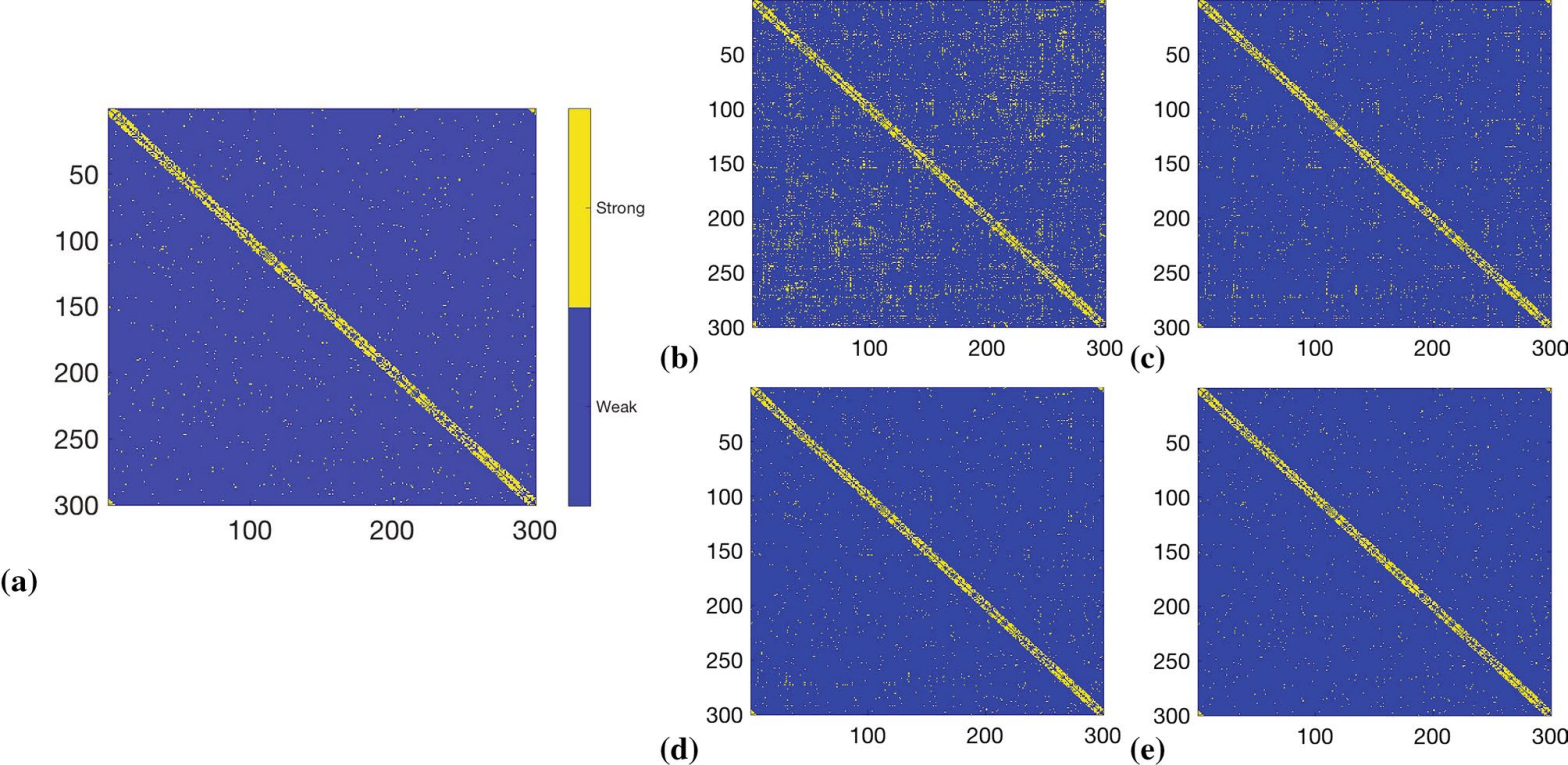
(**a**) Original topology of a sparse Kuramoto oscillator network. (**b**–**e**) Coupling strengths estimated for the sparse network using the PRP algorithm after 10, 30, 50, and 100 stages.

**Figure 5:**
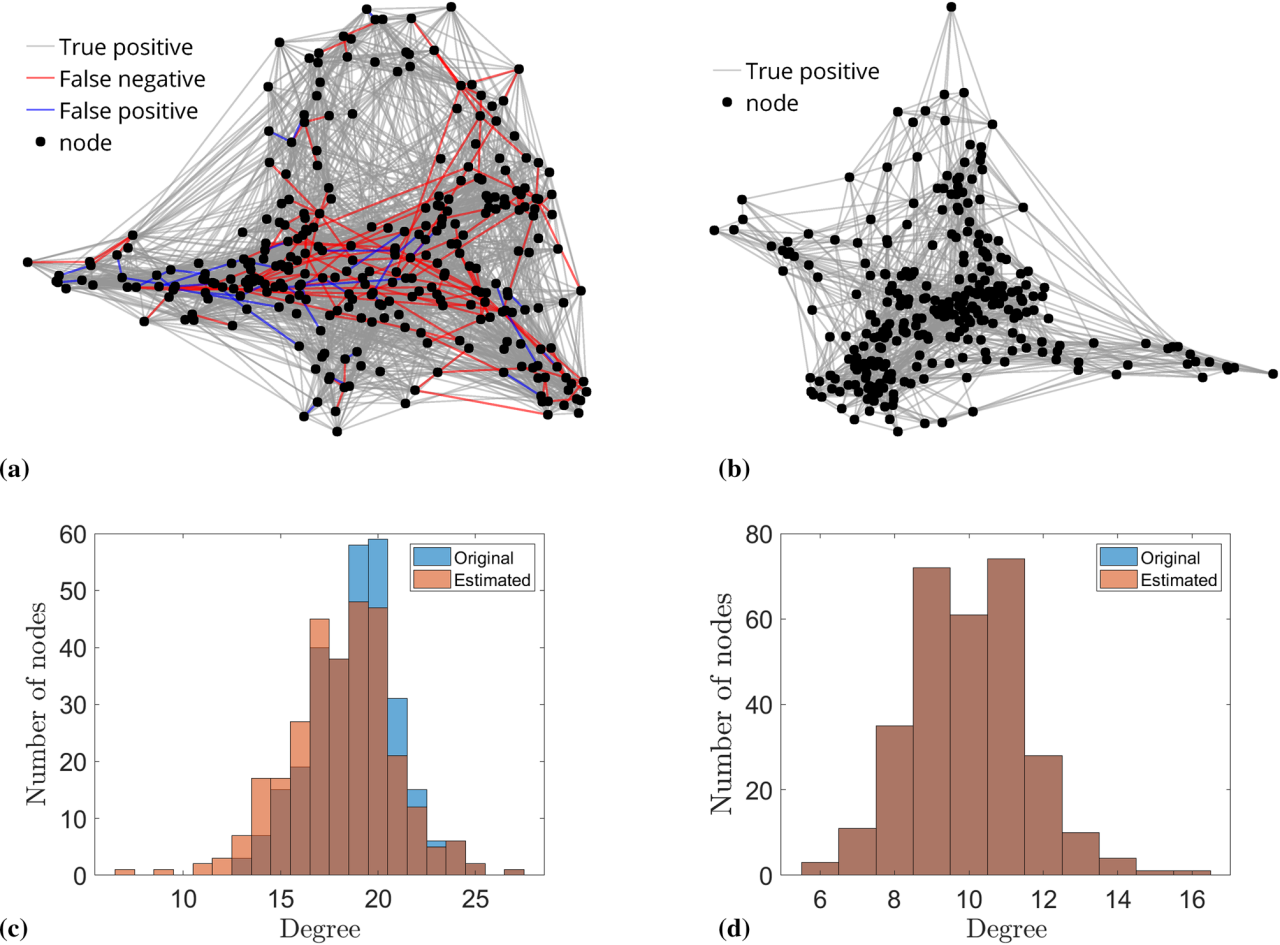
The Watts–Strogatz network model was used to generate a sparse Kuramoto oscillator network with $N = 300, K = 10, \beta = 0.3$; and $N = 300, K = 20, \beta = 0.3$ for a dense network. The frequency of Kuramoto oscillator was uniformly distributed in [5, 10]. The coupling strength was uniformly distributed in [0.4, 0.6] for a strong coupling; for a weak coupling, the coupling strength was set to 0 in the sparse network and 0.2 in the dense network. (**a**) Topology recovered for dense network after 450 stage using the PRP algorithm; true positive indicates a strong coupling recovered as strong one; false negative means a strong coupling recovered as weak one; false positive implies a weak coupling recovered as strong one; nodes without connections are considered as weakly coupled. (**b**) Topology recovered for sparse network after 100 stages using PRP the algorithm. (**c**) Degree histograms for the original and the estimated network after 450 stages for the dense Kuramoto oscillator network. Normalized Wasserstein distance of two histogram is $5.8\times 10^{-3}$. (**d**) Degree histograms for the original and the estimated network after 100 stages for the sparse network. Normalized Wasserstein distance of two histogram is 0, which indicates the network topology was perfectly recovered.

The estimation results regarding the strength of the interaction between two nodes for both dense and sparse network, across different number of stages, are presented in Figs. 3 and 4. The topology of the recovered dense and sparse networks of 300 nodes given 600,000 data points are reported in Fig. 5a, b, respectively. The results of the recovered topology after the PRP algorithm was terminated (450 stages for the dense network and 100 stages for the sparse network) are visualized in Fig. 5c, d and Table 1. It can be observed that for the dense network, after 450 stages of the PRP algorithm, 5,352 out of the 5,672 strong connections were correctly identified, while the remaining 320 were inferred as weak connections, yielding a $94.3\%$ accuracy to the network topology recovery; while for the sparse network, the recovery attained $100\%$ accuracy after 100 stages of PRP algorithm.

**Figure 6:**
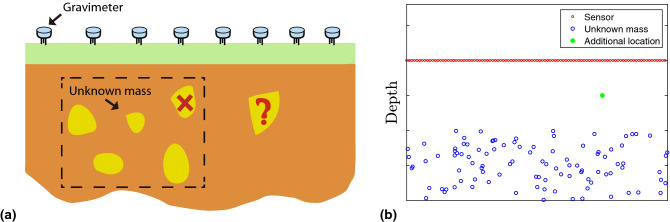
(**a**) Demonstration of estimating unknown masses in the subsurface using gravimeters. Suppose there are five objects (shown in the dashed block) whose locations and an estimate of their masses are known apriori, and if an additional object is detected, then estimating the masses all six objects can be formulated as an incremental problem. Alternatively, if one of the objects is proved to be absent, estimating the remaining four masses can be formulated as decremental problem. (**b**) Locations of gravimeters and unknown masses in the numerical experiments. Red circles indicate location of gravimeters, which are uniformly distributed on the surface; blue circles show where the unknown masses are in the subsurface; green dot represents the location where additional mass is detected. For ease of exposition, only 100 gravimeters and 100 unknown masses are shown in this figure.

Example 3: Gravimetric survey To illustrate the scalability of the PRP and its flexibility to handle incremental/decremental problems associated with an LIP, we present an application example using a gravimetric survey experiment. In geophysics, due to the inhomogeneity of lithology in the subsurface of the Earth, the gravity measured on the surface varies across different locations. Such perturbations on the gravitational field can be used to infer the mineralogical composition underground, which is formulated as LIPs based on the observed data^8, 34^. Here, we consider the problem of detecting large masses in the subsurface using the measured gravitational field, which can be encoded into an LIP. We demonstrate the flexibility of the PRP algorithm to solve this LIP. In particular, we observe that detecting additional mass and re-computing mass distribution in this application can be formulated as incremental/decremental problems. We generated a 2-D example for illustrating a gravimetric survey experiment (see Fig. 6a).

In the example, we considered a gravimetric survey experiment with 20,000 gravimeters to detect the existence of 10,000 unknown masses, as depicted in Fig. 6b. Suppose the locations and masses of 9,999 objects are already known. Then the estimation of the remaining object’s mass becomes an incremental LIP problem that requires solving an LIP of size $20{,}000 \times 10{,}000$, which cost 181.33 s when it was solved as a new problem using the PRP. However, it only consumed 9.8036 s for the PRRP algorithm (with $p=2$) to provide a solution when this incremental problem was adaptively updated using the existing solution corresponding to the LIP with 9,999 known objects, which significantly reduced the computations. The implementation details and analysis for this example are provided in the Supplementary Information, Section S.6.3.

**Table 1:** Topology recovered for dense (after 450 stages) and sparse network (after 100 stages) using the PRP algorithm.

Original	Recovered	
	Strong	Weak
**Dense network**		
Strong	5352	320
Weak	80	84,248
**Sparse network**		
Strong	3000	0
Weak	0	87,000

## Discussion

To summarize, we develop a parallel computational framework for solving large-scale LIPs and demonstrated its efficacy and flexibility using numerical examples from diverse scientific fields. In the following, we discuss some important features of the PRP algorithm, the design choices for implementing the PRP and their associated trade-offs, and how we deal with them in our numerical experiments.

The PRP algorithm reduces the computational resource required to solve a large-scale LIP by decomposing the given LIP into sub-problems and then fusing the solutions of these sub-problems using the proposed update rule in (2) to compute the solution of the given LIP. This is done by partitioning the matrix *A* into several column blocks $A_1, \ldots , A_p$. Then each block $A_i, i=1, \ldots , p$, is distributed to a computing agent to solve a smaller LIP associated with $A_i$. As illustrated through Example 1, finer partitions do not necessarily lead to faster convergence (see Fig. 2). The number of partitions and the size of each partition are design choices. We do not provide rules on how to partition *A* in order to achieve the ‘best’ performance since no computing agent has full-information of *A*. In our experiments, we empirically chose *p* and then evenly divided the columns of *A* into *p* column blocks. In addition to the partition of *A*, one needs to select the weights, i.e., $w_i$, of the residual for each sub-problem in order to compute the residual for the next stage using the update rule in (2). It is worthwhile to mention that the choice of $w_i$’s will influence the speed of convergence up to a constant factor (see (S.21) in Supplementary Information). The optimal choice of $w_i$’s yielding the fastest convergence cannot be obtained without calculating the eigen-decomposition of *A*. Therefore, in our numerical implementation, we picked $w_i = \frac{1}{p}, i = 1, \ldots , p$, as we have proved that the sequence of residuals $\{{\hat{R}}^{(k)}\}$ computed by (2) converges to the minimal residual of the original LIP exponentially regardless the choice of $w_i$’s (see Section S.2 in Supplementary Information). We also show that equipped with the RRP algorithm, the PRP framework is upgraded to the PRRP algorithm, which reduces the time needed to solve a large-scale LIP by parallelizing the RRP algorithm. Moreover, we show that the PRRP algorithm efficiently handles the incremental/decremental problems associated with an LIP. In particular, we provide a sufficient condition in (3) guaranteeing that PRRP expedites the process of solving the incremental/decremental problems, compared with the RRP algorithm. We also observe that the sufficient condition in (3) was satisfied in most of our experiments, though a rigorous guideline on how to choose the partitions $A_i$ that satisfy this sufficient condition is not provided due to the computational intractability.

## Conclusions

A seemingly inconspicuous problem like the LIP has a significant impact on scientific computing. Notwithstanding the plenitude of methods available for solving an LIP, several new challenges have emerged due to sheer volume of data, inadequate computational resources, security and privacy concerns, and the prevalence of relevant incremental/decremental problems. Inevitably, given such constraints, scalable, and distributed algorithms for solving exaggerated LIPs are much sought after. The pliability of the PRP framework enables the integration and expedition of any existing algorithm developed for directly solving a standard LIP. In particular, we show that the RRP algorithm, a state-of-the-art iterative algorithm for solving LIPs, can be accelerated in tandem with the PRP framework, which saved nearly $50\%$ of the computational time as recorded in our numerical experiments. Although rigorous guidelines on how to tailor the parameters in the PRRP algorithm to achieve the best performance are not provided due to computational intractability, we propose sufficient conditions that warrant faster convergence of this algorithm. We further provide feasible choices of design parameters for implementing the proposed PRP algorithm to solve any given large-scale LIP. The applications to complex problems, including the network inference and the gravimetric survey (see Sections S.6.2 and S.6.3 in Supplementary Information), illustrate the promise of the developed PRP algorithm to be equipped with desirable properties that can confront the existing challenges.

## Supplementary information

Supplementary Information.

## Data Availability

All data generated or analyzed during this study are included in this published article (and its Supplementary Information files).
