# Supplementary material for: Parallel residual projection: a new paradigm for solving linear inverse problems
Source: Sci Rep. 2020 Jul 30;10:12846. doi: 10.1038/s41598-020-69640-5 (PMC7393146; doi:10.1038/s41598-020-69640-5)
Supplement: Supplementary file 1 — Supplementary Information. [file 41598_2020_69640_MOESM1_ESM.pdf]

SI : Parallel Residual Projection - A New Paradigm for Solving Linear Inverse Problems

Wei Miao,[1] Vignesh Narayanan,[1] and Jr-Shin Li[1*]

[1]Department of Electrical and Systems Engineering, Washington University in St. Louis,
1 Brookings Drive, St. Louis, MO 63130, USA
[*]To whom correspondence should be addressed; E-mail: jsli@wustl.edu.

S.1 Introduction

We consider the linear inverse problem (LIP) of the form,

$$Ax = b, \tag{S.1}$$

where $A \in \mathbb{R}^{m\times n}$ is the regression map (coefficient matrix), $b \in \mathbb{R}^m$ is the vector of observations, and $x \in \mathbb{R}^n$ is the vector of unknown coordinates (variables). In many practical applications, the size of the LIP may vary with respect to the number of variables or available observations.

Incremental and Decremental Problems. For example, after a solution to a problem, say $A_1x = b_1$, is computed, as a new set of data (A_2, b_2) becomes available, the coordinate vector x will be revised by solving the augmented problem (referred to as the row incremental problem),

$$\begin{bmatrix} A_1 \\ A_2 \end{bmatrix} x = \begin{bmatrix} b_1 \\ b_2 \end{bmatrix}. \tag{S.2}$$

Similarly, after a solution to the problem in (S.2) is computed, if we have to delete a set of data (A_2, b_2), the vector x will be revised so that the new solution is the least-squares solution of the

decremental problem (row decrement),

$$A_1 x = b_1. \tag{S.3}$$

Analogously, after a solution to the problem in (S.3) is computed, when new columns, denoted as A_2, are appended to the matrix A_1, the coordinate vector x will be modified by solving the augmented incremental problem (column increment) with expanded columns,

$$\begin{bmatrix} A_1 & A_2 \end{bmatrix} \begin{bmatrix} x_1 \\ x_2 \end{bmatrix} = b_1, \tag{S.4}$$

where x_1 is the solution of the problem in (S.3).

Finally, after a solution to the problem in (S.4) is computed, and if some of the columns, say A_2, are required to be deleted, the coordinate vector x will be modified by solving the problem,

$$A_1 x = b_1, \tag{S.5}$$

resulting in the decremental problem (column decrement).

S.2 Parallel residual projection (PRP) for LIPs

Let the matrix $A \in \mathbb{R}^{m \times n}$ in (S.1) be partitioned into p column blocks such that $A = [A_1 | \ldots | A_p]$, where $A_i \in \mathbb{R}^{m \times d_i}$ and $\sum_{i=1}^{p} d_i = n$. Then the corresponding unknown coefficient vector is represented by $x = \begin{bmatrix} X_1 \\ \vdots \\ X_p \end{bmatrix}$ and the observation b is written as a sum of vectors, i.e., $b = \sum_{i=1}^{p} b_i$, where $X_i \in \mathbb{R}^{d_i}$ and $b_i \in \mathbb{R}^m$. Note that such a decomposition of b is not unique. Using these partitions, the problem in (S.1) is decomposed into p sub-problems of the form $A_i X_i = b_i$ with $i = 1, \ldots, p$. Instead of solving these p sub-problems directly, we will solve their related intermediate residual problems in multiple stages. In particular, at stage k, the residual problems are given by $A_i \, \delta X_i^{(k)} = R_i^{(k)}$, where $\delta X_i^{(k)}$ denotes the unknown variable and $R_i^{(k)}$ denotes the residual of sub-problem i at stage k with $R_i^{(0)} = b_i$ for $i = 1, \ldots, p$.

At each stage, the p residual problems are solved in parallel by p agents, and the resulting solution-residual pairs are used to update the solution of the original problem in (S.1) and initialize the residual of the intermediate residual problems for the next stage. Specifically, for each of the residual problems, we denote the solution-residual pair as $(\widehat{\delta X}_i^{(k)}, \hat{R}_i^{(k)})$. We then propose to initialize the residual for the intermediate problem at the beginning of $(k+1)^{th}$ stage as

$$R_i^{(k+1)} = w_i \hat{R}^{(k)} = w_i \sum_{j=1}^{p} \hat{R}_j^{(k)}, \tag{S.6}$$

with $w_i \in (0,1)$ satisfying $\sum_{i=1}^{p} w_i = 1$, and update the solution of (S.1) at the end of the $(k+1)^{th}$ stage as

$$\hat{X}_i^{(k+1)} = \hat{X}_i^{(k)} + \widehat{\delta X}_i^{(k)}, \tag{S.7}$$

for $i = 1, \dots, p$ and $k = 0, 1, \dots$ with the initial condition $\hat{X}^{(0)}$ and $\hat{R}^{(0)}$ satisfying $\hat{R}^{(0)} = b - A\hat{X}^{(0)}$.

The PRP algorithm introduced in this section to solve the LIP in (S.1), equipped with the proposed update rules as in (S.7) and (S.6), converges exponentially (see Theorem S.1). The pseudocode for the PRP algorithm is shown as Algorithm S.1.

We first present two lemmas that will be used to analyze the properties of the PRP algorithm.

Lemma S.1. *For any $A \in \mathbb{R}^{m\times n}$, the following statements hold:*

(i) $I - A(A'A)^{-1}A'$ is a positive semi-definite matrix, where I is the $m \times m$ identity matrix,

(ii) $\|I - A(A'A)^{-1}A'\|_2 = 1$.

Proof. (i) Let $P = I - A(A'A)^{-1}A'$. Then, we have $P'P = PP' = P^2 = P$, and thus $x'Px = x'P^2x = (Px)'(Px) \geq 0$ for any $x \in \mathbb{R}^m$, which implies that P is positive semi-definite.

(ii) From the definition of the matrix 2-norm, we observe that $\|Px\|_2^2 = \langle Px, Px\rangle = x'P^2x = x'Px = \langle Px, x\rangle \leq \|Px\|_2\|x\|_2$. Hence we have $\|Px\|_2 \leq \|x\|$. This leads to

$$\|P\|_2 \leq 1. \tag{S.8}$$

On the other hand, we have $\|P\|_2 = \|P \cdot P\|_2 \leq \|P\|_2\|P\|_2$, which gives

$$\|P\|_2 \geq 1. \tag{S.9}$$

Combining (S.8) and (S.9) yields $\|P\|_2 = 1$. □

Lemma S.2. *Let $A \in \mathbb{R}^{m\times n}$ be a full column rank matrix partitioned as $A = [A_1|\cdots|A_p]$ and $B = I - \sum_{i=1}^p w_i A_i \left(A_i'A_i\right)^{-1} A_i'$, where $w_i \in [0,1]$ with $\sum_{i=1}^p w_i = 1$. Then the following statements are true:*

(i) All eigenvalues of B lie in the interval $[0,1]$.

(ii) If $E_1(B)$ denotes the eigenspace of B spanned by eigenvectors corresponding to the eigenvalue 1, then $E_1(B) = \mathcal{R}(A)^\perp$.

Proof. (i) For any $x \in \mathbb{R}^m$, we have

$$\begin{aligned} x'Bx &= x'\left(I - \sum_{i=1}^p w_i A_i(A_i'A_i)^{-1}A_i'\right)x \\ &= \sum_{i=1}^p w_i x'(I - A_i(A_i'A_i)^{-1}A_i')x \geq 0, \end{aligned} \tag{S.10}$$

by Lemma S.1 and non-negativity of w_i. Therefore, B is positive semidefinite and its eigenvalues are lower bounded by 0. Next, we calculate the upper bound for the eigenvalues of B. From the definition of B, we have

$$\begin{aligned} \|B\|_2 &= \|I - \sum_{i=1}^p w_i A_i(A_i'A_i)^{-1}A_i'\|_2 = \|\sum_{i=1}^p w_i(I - A_i(A_i'A_i)^{-1}A_i')\|_2 \\ &\leq \sum_{i=1}^p w_i\|I - A_i(A_i'A_i)^{-1}A_i'\|_2 = \sum_{i=1}^p w_i = 1, \end{aligned} \tag{S.11}$$

by Lemma S.1. Because B is symmetric, $\|B\|_2 = \lambda_{max}(B'B) = \lambda_{max}(B^2) = (\lambda_{max}(B))^2$, where $\lambda_{max}(B)$ denotes the largest eigenvalue of B. This together with (S.11) gives $\lambda_{max}(B) \leq 1$. Therefore, all eigenvalues of B lies in $[0, 1]$.

(ii) For any $x \in E_1(B)$, we have $[I - \sum_{i=1}^{p} w_i A_i (A_i' A_i)^{-1} A_i']\, x = x$, which reveals that $\sum_{i=1}^{p} \hat{A}_i x = 0$, where $\hat{A}_i = A_i (A_i' A_i)^{-1} A_i'$. Note that since $\hat{A}_i$ is a projection matrix (i.e., $\hat{A}_i' = \hat{A}_i$ and $\hat{A}_i^2 = \hat{A}_i$), $\hat{A}_i x \in \mathcal{R}(A_i)$ for each $i \in \{1, \ldots, p\}$. As the matrix A is full column rank, $\mathcal{R}(A_i)$'s are linear independent so that $\sum_{i=1}^{p} \hat{A}_i x = 0$, implying $\hat{A}_i x = 0$ for all $i = 1, \ldots, p$, which further implies that $x \in \mathcal{R}(A)^{\perp}$, and hence, we have

$$E_1(B) \subseteq \mathcal{R}(A)^{\perp}. \tag{S.12}$$

On the other hand, for any $x \in \mathcal{R}(A)^{\perp}$, $Bx = [I - \sum_{i=1}^{p} w_i A_i (A_i' A_i)^{-1} A_i']\, x = x - 0 = x$. Therefore, $x \in E_1(B)$, and hence, we have

$$\mathcal{R}(A)^{\perp} \subseteq E_1(B). \tag{S.13}$$

From (S.12) and (S.13), we conclude that $E_1(B) = \mathcal{R}(A)^{\perp}$. Note that this conclusion holds when 1 is not an eigenvalue of B. In this case, $E_1(B) = \{\mathbf{0}\}$ and $\mathcal{R}(A) = \mathbb{R}^m$, where $\mathbf{0}$ is the m-dimensional zero vector. □

Theorem S.1 (PRP algorithm)**.** *Consider the LIP given by $Ax = b$, where $A = [A_1 | \cdots | A_p] \in \mathbb{R}^{m \times n}$ is of full column rank with $A_i \in \mathbb{R}^{m \times d_i}$ and $\sum_{i=1}^{p} d_i = n$. Let $\hat{X}^{(k)} = \begin{bmatrix} \hat{X}_1^{(k)} \\ \vdots \\ \hat{X}_p^{(k)} \end{bmatrix}$ and $\hat{R}^{(k)} = \sum_{i=1}^{p} \hat{R}_i^{(k)}$ be the respective solution estimate and residual of the LIP generated by the PRP algorithm (Algorithm S.1) at the end of stage k using the update rules as in* (S.6) *and* (S.7)*, with the initial condition $\hat{X}^{(0)}$ and $\hat{R}^{(0)}$ satisfying $\hat{R}^{(0)} = b - A\hat{X}^{(0)}$. Then,*

(i) there exists a constant $C > 0$ and $\lambda \in (0, 1)$ such that $\|\hat{R}^{(k)} - \hat{R}\|_2 \leq C\lambda^k$, where $\hat{R}$ is the minimum residual of the LIP; and

(ii) $\lim_{k \to \infty} \hat{X}^{(k)} \to \hat{X}$, where $\hat{X}$ is the least-squares solution of the LIP.

Proof. (i) The minimum residual of the ith residual problem at stage k satisfies

$$\hat{R}_i^{(k)} = \left[I - A_i\left(A_i'A_i\right)^{-1}A_i'\right]R_i^{(k)}. \tag{S.14}$$

From (S.14), we have

$$\sum_{i=1}^{p}\hat{R}_i^{(k)} - \hat{R} = \sum_{i=1}^{p}\left\{\left[I - A_i\left(A_i'A_i\right)^{-1}A_i'\right]R_i^{(k)}\right\} - \hat{R}. \tag{S.15}$$

Using (S.6), we can simplify (S.15) to get

$$\begin{aligned}\sum_{i=1}^{p}\hat{R}_i^{(k)} - \hat{R} &= \sum_{i=1}^{p}\left\{\left[I - A_i\left(A_i'A_i\right)^{-1}A_i'\right]\sum_{j=1}^{p}w_i\hat{R}_j^{(k-1)}\right\} - \hat{R}\\ &= \left(\sum_{j=1}^{p}\hat{R}_j^{(k-1)} - \hat{R}\right) - \sum_{i=1}^{p}w_iA_i\left(A_i'A_i\right)^{-1}A_i'\sum_{j=1}^{p}\hat{R}_j^{(k-1)}. \end{aligned}\tag{S.16}$$

Observe that the minimum residual satisfies $\sum_{i=1}^{p}A_i\left(A_i'A_i\right)^{-1}A_i'\hat{R} = 0$. Therefore, (S.16) can be modified to get

$$\begin{aligned}\sum_{i=1}^{p}\hat{R}_i^{(k)} - \hat{R} &= \left(\sum_{j=1}^{p}\hat{R}_j^{(k-1)} - \hat{R}\right) - \sum_{i=1}^{p}w_iA_i\left(A_i'A_i\right)^{-1}A_i'\left(\sum_{j=1}^{p}\hat{R}_j^{(k-1)} - \hat{R}\right)\\ &= \left[I - \sum_{i=1}^{p}w_iA_i\left(A_i'A_i\right)^{-1}A_i'\right]\left(\sum_{i=1}^{p}\hat{R}_i^{(k-1)} - \hat{R}\right). \end{aligned}\tag{S.17}$$

For ease of exposition, let $B = I - \sum_{i=1}^{p}w_iA_i\left(A_i'A_i\right)^{-1}A_i'$ and $E^{(k)} = \sum_{i=1}^{p}\hat{R}_i^{(k)} - \hat{R}$. Then, (S.17) can be rewritten as $E^{(k)} = BE^{(k-1)}$. Solving this linear difference equation with the initial condition $\hat{X}^{(0)}$ and $\hat{R}^{(0)}$ reveals that

$$E^{(k)} = B^kE^{(0)} = B^k(\hat{R}^{(0)} - \hat{R}). \tag{S.18}$$

From the results of Lemma 2, the eigenvalues of B are in the interval $[0, 1]$, and as a consequence, (S.18) describes a contracting map.

Suppose matrix B has eigenvalue 1 with multiplicity d, since B is real and symmetric, there exists an eigen-decomposition for the matrix B such that $B = PDP'$ where

$$P = [P_1, \ldots, P_d, P_{d+1}, \ldots, P_m],$$

is an orthogonal matrix, and

$$D = \text{diag}\,(1, \ldots, 1, \lambda_{d+1}, \ldots, \lambda_m),$$

is a diagonal matrix with the eigenvalues $1 > \lambda_{d+1} \geq \cdots \geq \lambda_m \geq 0$. By Lemma S.2, span $\{P_1, \ldots, P_d\} = E_1(B) = \mathcal{R}(A)^\perp$. Therefore, we have span $\{P_{d+1}, \ldots, P_m\} = \mathcal{R}(A)$. Since $\hat{R}^{(0)} - \hat{R} \in \mathcal{R}(A)$, there exists scalars $s_i \in \mathbb{R}$ for $i = 1, \ldots, m$ such that

$$\hat{R}^{(0)} - \hat{R} = \sum_{i=d+1}^{m} s_i P_i. \tag{S.19}$$

By substituting (S.19) into (S.18), we get

$$\begin{aligned} E^{(k)} = B^k(\hat{R}^{(0)} - \hat{R}) &= \sum_{i=d+1}^{m} s_i B^k P_i, \\ &= \sum_{i=d+1}^{m} s_i \lambda_i^k P_i. \end{aligned} \tag{S.20}$$

Applying the norm operator on both sides of (S.20) yields,

$$\|E^{(k)}\|_2 = \| \sum_{i=d+1}^{m} s_i \lambda_i^k P_i \| \leq \sum_{d+1}^{m} |s_i| \lambda_i^k \leq \sum_{d+1}^{m} |s_i| (\max_i(\lambda_i))^k \leq C\lambda^k, \tag{S.21}$$

where $C = \sum_{i=d+1}^{m} |s_i|$ and $\lambda = \max_i(\lambda_i) \in (0, 1)$. Therefore, $\|\hat{R}^{(k)} - \hat{R}\|_2 \to 0$ as $k \to \infty$, which implies $\lim_{k\to\infty} \hat{R}^{(k)} = \hat{R}$.

(ii) From the initial conditions, $\hat{R}^{(k)} = b - A\hat{X}^{(k)}$ holds for $k = 0$. We prove that $\hat{R}^{(k)} = b - A\hat{X}^{(k)}$ by induction for all $k \in \mathbb{N}$. Suppose $\hat{R}^{(k-1)} = b - A\hat{X}^{(k-1)}$ holds. Now, by definition of $\hat{R}_i^{(k)}$, we have

$$\hat{R}_i^{(k)} = R_i^{(k)} - A_i \widehat{\delta X}_i^{(k)}, i = 1, \ldots, p. \tag{S.22}$$

Taking summation on both sides of (S.22) yields

$$\sum_{i=1}^{p} \hat{R}_i^{(k)} = \sum_{i=1}^{p} R_i^{(k)} - A\widehat{\delta X}^{(k)}, \tag{S.23}$$

where $\widehat{\delta X}^{(k)} = \begin{bmatrix} \widehat{\delta X}_1^{(k)} \\ \vdots \\ \widehat{\delta X}_p^{(k)} \end{bmatrix}$. Substituting the update rule in (S.6) into (S.23), we get

$$\sum_{i=1}^{p} \hat{R}_i^{(k)} = \sum_{i=1}^{p} \hat{R}_i^{(k-1)} - A\widehat{\delta X}^{(k)} = \hat{R}^{(k-1)} - A\widehat{\delta X}^{(k)} \tag{S.24}$$

Substituting $\hat{R}^{(k-1)} = b - A\hat{X}^{(k-1)}$ in (S.24), we have

$$\sum_{i=1}^{p} \hat{R}_i^{(k)} = b - A\hat{X}^{(k-1)} - A\widehat{\delta X}^{(k)}. \tag{S.25}$$

Substituting the update equation in (S.7) into (S.25) reveals

$$\sum_{i=1}^{p} \hat{R}_i^{(k)} = b - A\hat{X}^{(k)} = \hat{R}^{(k)}. \tag{S.26}$$

Hence, $\hat{R}^{(k)} = b - A\hat{X}^{(k)}$ holds for $k = 0, 1, 2, \ldots$, which implies that $\lim_{k\to\infty} \hat{R}^{(k)} = \lim_{k\to\infty} b - A\hat{X}^{(k)} = \hat{R}$. By uniqueness of $\hat{X}$, we have $\lim_{k\to\infty} \hat{X}^{(k)} = \hat{X}$. $\square$

Remark : As shown in the proof of Theorem S.1, it is worth mentioning that the PRP algorithm remains exponentially convergent with respect to the stages regardless of how the p sub-problems are solved. In Section S.5, we show that equipped with an appropriate iterative approach for solving the sub-problems, the PRP can be configured to tackle the incremental/decremental problems in a computationally efficient manner.

Remark : As for the weights $w_1, \ldots, w_p$ in the PRP algorithm, although they affect the convergence of $X^{(k)}$ by influencing the $\max_i(\lambda_i)$ in (S.21), determining the optimal choice of weights is intractable since it requires computing the spectrum of A. Therefore, in the experiments presented in this paper, we choose $w_i = \frac{1}{p}, i = 1, \ldots, p$.

The number of stages required to achieve ϵ- accuracy for the residual and the solution coordinate using the PRP algorithm is summarized next.

Theorem S.2. *The total number of stages needed for the PRP algorithm (Algorithm S.1) to achieve ϵ-accuracy for the residual, i.e., $\|\hat{R}^{(k)} - \hat{R}\|_2 \leq \epsilon$, is given by $\left[\log_\lambda \frac{\epsilon}{C}\right] + 1$, where $\left[\log_\lambda \frac{\epsilon}{C}\right]$ denotes the largest integer smaller than or equal to $\log_\lambda \frac{\epsilon}{C}$, and C and λ are the constants defined as in* (S.21). *In addition, if A is full column rank, then the total number of stages needed to achieve ϵ-accuracy for the solution, i.e. $\|\hat{X}^{(k)} - \hat{X}\|_2 < \epsilon$, is given by $\left[\log_\lambda \frac{\|A^\dagger\|_2 \epsilon}{C}\right] + 1$, where $A^\dagger$ is the Moore—Penrose inverse of A.*

Proof. The proof for residual follows directly from (S.21). For the solution accuracy, when A is full column rank, we have

$$\|\hat{X}^{(k)} - \hat{X}\| \leq \frac{1}{\|A^\dagger\|_2} \|\hat{R}^{(k)} - \hat{R}\|_2 \leq \frac{1}{\|A^\dagger\|_2} C\lambda^k.$$

Therefore the number of stages to achieve ϵ-accuracy for the solution is given by $\left[\log_\lambda \frac{\|A^\dagger\|_2 \epsilon}{C}\right] + 1$. □

Algorithm S.1 Parallel residual projection (PRP)

function PRP(A, b, p)
  Initialize: $A = [A_1, \ldots, A_p]$, $A_i \in \mathbb{R}^{m \times d_i}$, $\hat{R}_i^{(0)} = b_i$, satisfying $\sum_{i=1}^p b_i = b$,
    $\hat{X}_i^{(0)} = 0$, $i = 1, \ldots, p$ and $w_1, \ldots, w_p$, such that $0 < w_i < 1$, $i = 1, \ldots, p$,
    and $\sum_{i=1}^p w_i = 1$.
  for $k \leftarrow 1, 2, \ldots$ **do**:
    $R_i = w_i \sum_{j=1}^p \hat{R}_j$
    parfor $i = 1, \ldots, p$ **do**
      $\widehat{\delta X}_i = \underset{\delta X}{\text{argmin}} \|R_i - A_i \delta X\|_2$
      $\hat{R}_i = R_i - A_i \widehat{\delta X}_i$
    end parfor
    $\hat{X}_i = \hat{X}_i + \widehat{\delta X}_i$
  end for
  return $x = \begin{bmatrix} \hat{X}_1 \\ \vdots \\ \hat{X}_p \end{bmatrix}$, $r = \sum_{i=1}^p \hat{R}_i$
end function

S.3 Randomized residual projection for LIPs

The randomized residual projection (RRP) algorithm is a randomized iterative method to solve an LIP. Though the RRP is already proposed as a variant of the 'coordinate descent' algorithm in the literature (*29*), the proofs of convergence rate and complexity analysis of the algorithm are not detailed. Therefore, after a brief description of the RRP algorithm, we derive the convergence rate and present the complexity analysis for the RRP.

We denote $\hat{x}$, $\hat{r}$ as the least-squares solution, minimum residual of the LIP in (S.1), respectively, and $\mathcal{R}(A)$ as the column space of the matrix A. The RRP algorithm starts with an initialization of the residual, i.e., $r^{(0)} = b - Ax^{(0)}$, by choosing an arbitrary initial solution $x^{(0)}$. Following the same idea as in (*29*), at the $(k+1)^{\text{th}}$ iteration, we select an index $I(k+1) \in \{1, \dots, n\}$ and then the $(I(k+1))^{\text{th}}$ column of A, denoted by $a_{I(k+1)}$. The residual $r^{(k)}$, from the previous iteration, is then projected onto the column $a_{I(k+1)}$ to update the $(I(k+1))^{\text{th}}$ component of the solution vector, i.e., $x^{(k)}_{I(k+1)} \to x^{(k+1)}_{I(k+1)}$ and then the residual is updated $r^{(k)} \to r^{(k+1)} := b - Ax^{(k)}$. Following this procedure, the residual is iteratively reduced and asymptotically becomes orthogonal to all of the columns of A. This steers the sequence $\{x^{(k)}\}$ to the least-squares solution. To bring the residual orthogonal to the $\mathcal{R}(A)$ expeditiously, inspired by the randomized Kaczmarz algorithm (*30*), the index $I(k+1) \in \{1, \dots, n\}$ is selected randomly with the probability proportional to the squared norm of the selected column of A, i.e., $\|a_{I(k+1)}\|_2^2$. The pseudo code of the RRP algorithm is provided in Algorithm S.2.

Theorem S.3 (The Randomized Residual Projection Algorithm)**.** *Consider the linear inverse problem $Ax = b$, where $A \in \mathbb{R}^{m \times n}$ is of full column rank, $x \in \mathbb{R}^n$, and $b \in \mathbb{R}^m$. Starting from an initial solution $x^{(0)}$ with the resulting initial residual*

$$r^{(0)} = b - Ax^{(0)}, \tag{S.27}$$

the solution $x^{(k)}$ obtained by the iteration equations based on randomized residual projections,

given by

$$x_j^{(k+1)} = \begin{cases} x_j^{(k)} + \frac{a'_{I(k+1)} r^{(k)}}{\|a_{I(k+1)}\|_2^2}, & \textit{if } I(k+1) = j \\ x_j^{(k)}, & \textit{if } I(k+1) \neq j, \end{cases} \tag{S.28}$$

$$r^{(k+1)} = r^{(k)} - \frac{a'_{I(k+1)} r^{(k)}}{\|a_{I(k+1)}\|_2^2} a_{I(k+1)}, \tag{S.29}$$

converges to the least-squares solution asymptotically as $k \to \infty$*, where* $x_j^{(k)}$ *is the* j^{th} *element of* $x_j^{(k)}$*, and* $a_{I(k+1)}$ *is the* $I(k+1)^{th}$ *column of* A *that is randomly selected with the probability proportional to* $\|a_{I(k+1)}\|_2^2$.

Proof. The proof of this theorem is presented in three steps: (i) we show that the sequence of the norm of residuals $\{\|r^{(k)}\|_2\}_{k=0}^{\infty}$ is a convergent sequence; (ii) we show that the sequence $\{\|r^{(k)}\|_2\}_{k=0}^{\infty}$ converges to the norm of minimum residual $\|\hat{r}\|_2$; and (iii) finally, we prove that the sequence $\{x^{(k)}\}_{k=0}^{\infty}$ and residuals $\{r^{(k)}\}_{k=0}^{\infty}$ converge to the least squares solution $\hat{x}$ and minimum residual $\hat{r}$, respectively, under the update rule given in (S.28) when A is full column rank.

(i) Using the definition of the residual, at the $(k+1)^{\text{th}}$ iteration, we have

$$r^{(k+1)} = b - Ax^{(k+1)} = b - \sum_{j=1}^{n} a_j x_j^{(k+1)}. \tag{S.30}$$

Algorithm S.2 Randomized residual projection (RRP)

function RRP(A, x_0, r_0)
  Initialize: $x = x_0, r = r_0$
  for $k \leftarrow 1, 2, \ldots$ **do**
    Pick $I = j$ **with probability** $p_j = \frac{\|a_j\|_2^2}{\|A\|_F^2}$
    $\delta = \frac{\langle a_I, r \rangle}{\|a_I\|_2^2}$
    $x_I = x_I + \delta$
    $r = r - a_I \delta$
  end for
  return x, r
end function

Utilizing the update rules given in (S.28), (S.30) is expanded as

$$r^{(k+1)} = b - \sum_{j=1}^{n} a_j x_j^{(k)} - a_{I(k+1)} \left(x_{I(k+1)}^{(k+1)} - x_{I(k+1)}^{(k)} \right).$$

From (S.29),

$$r^{(k+1)} = \left(I - \frac{a_{I(k+1)} a'_{I(k+1)}}{\|a_{I(k+1)}\|_2^2} \right) r^{(k)} \doteq \left(I - P_{k+1} \right) r^{(k)}. \tag{S.31}$$

Observe that P_{k+1} is a projection matrix, and hence, from (S.31), we have

$$\|r^{(k+1)}\|_2 \leq \|r^{(k)}\|_2, \quad \forall\, k \in \mathbb{N}, \tag{S.32}$$

and the equality holds if and only if $r^{(k)} \perp a_{I(k+1)}$. Furthermore, the sequence $\left\{ \|r^{(k)}\|_2 \right\}_{k=0}^{\infty}$ is bounded below by the norm of the minimum residual, i.e., $\|\hat{r}\|_2 \leq \|r^{(k)}\|_2, \forall k \in \mathbb{N}$. Therefore, it can be concluded by the monotone convergence theorem that the sequence, $\left\{ \|r^{(k)}\|_2 \right\}_{k=0}^{\infty}$, (non-increasing and bounded below) is a convergent sequence.

(ii) To prove that $\lim_{k \to \infty} \|r^{(k)}\|_2 = \|\hat{r}\|_2$, we demonstrate that there exists a subsequence to the sequence $\{\|r^{(k)}\|_2\}_{k=0}^{\infty}$ that converges to $\|\hat{r}\|_2$.

Pick a subsequence $\left\{ y^{(p)} \right\}_{p=1}^{\infty} = \left\{ r^{(k_p)} \right\}_{p=1}^{\infty} \subseteq \left\{ r^{(k)} \right\}_{k=0}^{\infty}$, where $k_p < k_{p+1}, \forall p \in \mathbb{N}$, such that at the k_p^{th} iteration, the column index of the matrix A selected for projection satisfies $I(k_p) = (p \mod n) + 1$, i.e., $I(k_0) = 1,\ I(k_1) = 2,\ \ldots,\ I(k_{n-1}) = n,\ I(k_n) = 1,\ I(k_{n+1}) = 2, \ldots$. From the sequence $\left\{ y^{(p)} \right\}_{p=0}^{\infty}$, we construct $\left\{ z^{(p)} \right\}_{p=0}^{\infty} = \left\{ y^{(np)} \right\}_{p=0}^{\infty} \subset \left\{ y^{(p)} \right\}_{p=0}^{\infty}$. Therefore, by construction, z_{p+1} is obtained by projecting z_p on spaces spanned by each column of A at least once. If there exists a column of A, say a_I, such that $z^{(p)} \not\perp a_I$, then we have $\|z^{(p+1)}\|_2 < \|z^{(p)}\|_2$. Since $\|\hat{r}\|_2 \leq \|z_2^{(p)}\|, \forall p \in \mathbb{N}$, by the monotone convergence theorem, $\left\{ \|z^{(p)}\|_2 \right\}_{p=0}^{\infty}$ converges to $\|\hat{r}\|_2$, and as a consequence, we have

$$\lim_{k \to \infty} \|r^{(k)}\|_2 = \lim_{p \to \infty} \|z^{(p)}\|_2 = \|\hat{r}\|_2. \tag{S.33}$$

(iii) Finally, from (S.33), we have $\lim_{k\to\infty} \|b - Ax^{(k)}\|_2 = \|\hat{r}\|_2$. If A is full column rank, by the uniqueness of the least-squares solution, we can conclude that the sequence $\{x^{(k)}\}_{k=0}^{\infty}$ converges to the least-squares solution $\hat{x}$, under the update rule given in (S.28), which further implies $\{r^{(k)}\}_{k=0}^{\infty}$ converges to $\hat{r}$ under the update rule in (S.29). □

The random choice of column indices in the RRP algorithm under the update rules given in (S.28) and (S.29) expedite the convergence of $\{r^{(k)}\}_{k=0}^{\infty}$, $\{x^{(k)}\}_{k=0}^{\infty}$. Specifically, the residual error is exponentially convergent (also referred as linear convergence rate) in expectation over the product distribution of $(I(1), \ldots, I(k))$. This result is summarized in the following Theorem.

For ease of exposition, we denote the conditional expectation of the residual error at $(k+1)^{\text{th}}$ iteration as $\mathbb{E}_{I(k+1)\big|\tilde{I}(k)}\left(\|r^{(k+1)} - \hat{r}\|_2^2\right) \doteq \mathbb{E}_{I(k+1)}\left(\|r^{(k+1)} - \hat{r}\|_2^2\Big|\tilde{I}(k)\right)$, $\tilde{I}(k)$ is used in place of $(I(1), \ldots, I(k))$, and the probability of the random variable I taking value s is denoted as $f_I(s) \doteq \mathbb{P}(I = s)$.

Theorem S.4. *Suppose that $\hat{x}$ is the least-squares solution of the linear inverse problem as in* (S.1) *and $\hat{r}$ is the corresponding minimum residual. Let $\{x^{(k)}\}_{k=0}^{\infty}$ and $\{r^{(k)}\}_{k=0}^{\infty}$ be the sequences generated by the RRP iterations as in* (S.28) *and* (S.29)*, respectively. Then, these sequences converge to $\hat{x}$ and $\hat{r}$, respectively, exponentially in expectation over the product distribution of $(I(1), \ldots, I(k))$, where $I(p), \forall p \in \{1, \ldots, k\}$ is a random variable taking values from $\{1, \ldots, n\}$, under a discrete distribution such that $\mathbb{P}(I(k) = j) = \frac{\|a_j\|_2^2}{\|A\|_F^2}$. Specifically we have*

$$\mathbb{E}_{\tilde{I}(k)}\|r^{(k)} - \hat{r}\|_2^2 \leq \left(1 - \frac{1}{\kappa^2(A)}\right)^k \|r^{(0)} - \hat{r}\|_2^2, \tag{S.34}$$

$$\mathbb{E}_{\tilde{I}(k)}\|x^{(k)} - \hat{x}\|_2^2 \leq \left(1 - \frac{1}{\kappa^2(A)}\right)^k \frac{\|r^{(0)} - \hat{r}\|_2^2}{\|A^{\dagger}\|_2^2}, \tag{S.35}$$

where $\kappa(A) \doteq \|A\|_F\|A^{-1}\|_2$ is the modified condition number of A, $\|A\|_F$ denotes the Frobenius norm of A, and $A^{\dagger}$ is the Moore—Penrose inverse of A.

Proof. From (S.29), we have

$$r^{(k+1)} = (I - P_{k+1})r^{(k)}, \tag{S.36}$$

where $P_{k+1} = \frac{a_{I(k+1)}a'_{I(k+1)}}{\|a_{I(k+1)}\|_2^2}$ is a random projection matrix computed based on the choice of the random variable $I(k+1)$. For all $k \in \mathbb{N}$, by definition of the Euclidean norm, we have

$$\|r^{(k+1)} - \hat{r}\|_2^2 = (r^{(k+1)} - \hat{r})'(r^{(k+1)} - \hat{r}). \tag{S.37}$$

By substituting (S.36) into (S.37), we have

$$\begin{aligned} \|r^{(k+1)} - \hat{r}\|_2^2 &= \big((I - P_{k+1})r^{(k)} - \hat{r}\big)'\big((I - P_{k+1})r^{(k)} - \hat{r}\big) \\ &= r'^{(k)}(I - P_{k+1})^2 r^{(k)} - 2\hat{r}'(I - P_{k+1})r^{(k)} + \hat{r}'\hat{r}. \end{aligned} \tag{S.38}$$

On simplification of (S.38) using the fact that $(I - P_{k+1})^2 = I - P_{k+1}$, we get

$$\begin{aligned} \|r^{(k+1)} - \hat{r}\|_2^2 &= r'^{(k)}(I - P_{k+1})r^{(k)} - 2\hat{r}'(I - P_{k+1})r^{(k)} + \hat{r}'\hat{r} \\ &= \|r^{(k)} - \hat{r}\|_2^2 - r'^{(k)}P_{k+1}r^{(k)}. \end{aligned} \tag{S.39}$$

Since the minimum residual $\hat{r}$ is perpendicular to column space of A, i.e., $\hat{r} \perp \mathcal{R}(A)$, we have, for any $k \in \mathbb{N}$, $\hat{r}'P_{k+1}r^{(k)} = 0$, and $\hat{r}'P_{k+1}\hat{r} = 0$. Therefore, we observe that

$$r'^{(k)}P_{k+1}r^{(k)} = (r^{(k)} - \hat{r})'P_{k+1}(r^{(k)} - \hat{r}). \tag{S.40}$$

Substituting (S.40) into (S.39) yields

$$\begin{aligned} \|r^{(k+1)} - \hat{r}\|_2^2 &= \|r^{(k)} - \hat{r}\|_2^2 - (r^{(k)} - \hat{r})'P_{k+1}(r^{(k)} - \hat{r}) \\ &= \|r^{(k)} - \hat{r}\|_2^2 \left[1 - \frac{(r^{(k)} - \hat{r})'P_{k+1}(r^{(k)} - \hat{r})}{\|r^{(k)} - \hat{r}\|_2^2}\right]. \end{aligned} \tag{S.41}$$

Since $r^{(k+1)}$ is a random variable uniquely determined by $\tilde{I}(k), I(k+1)$, computing the conditional expectation over $I(k+1)\big|\tilde{I}(k)$ on both sides of (S.41) yields

$$\mathbb{E}_{I(k+1)\big|\tilde{I}(k)}\left(\|r^{(k+1)} - \hat{r}\|_2^2\right) = \mathbb{E}_{I(k+1)\big|\tilde{I}(k)}\left(\|r^{(k)} - \hat{r}\|_2^2\left[1 - \frac{(r^{(k)} - \hat{r})'P_{k+1}(r^{(k)} - \hat{r})}{\|r^{(k)} - \hat{r}\|_2^2}\right]\right). \tag{S.42}$$

Notice that $I(k+1)$ is independent from $\tilde{I}(k)$. Therefore, by the definition of conditional expectation, (S.42) can be computed by

$$\mathbb{E}_{I(k+1)\big|\tilde{I}(k)}\left(\|r^{(k+1)}-\hat{r}\|_2^2\right) = \sum_{(r)} r\mathbb{P}(r^{(k+1)} = r\big|\tilde{I}(k)) = \sum_{(r)} r\mathbb{P}(r^{(k+1)} = r\big|r^{(k)})$$

$$= \sum_{j=1}^{n} \mathbb{P}(I(k+1)=j)\|r^{(k)}-\hat{r}\|_2^2 \left[1 - \frac{(r^{(k)}-\hat{r})'\frac{a_j a_j'}{\|a_j\|_2^2}(r^{(k)}-\hat{r})}{\|r^{(k)}-\hat{r}\|_2^2}\right] \quad \text{(S.43)}$$

Substituting $\mathbb{P}((I(k+1)=j) = \frac{\|a_j\|_2^2}{\|A\|_F^2}$ into (S.43) yields

$$\mathbb{E}_{I(k+1)\big|\tilde{I}(k)}\left(\|r^{(k+1)}-\hat{r}\|_2^2\right) = \sum_{j=1}^{n} \frac{\|a_j\|_2^2}{\|A\|_F^2}\|r^{(k)}-\hat{r}\|_2^2 \left[1 - \frac{(r^{(k)}-\hat{r})'\frac{a_j a_j'}{\|a_j\|_2^2}(r^{(k)}-\hat{r})}{\|r^{(k)}-\hat{r}\|_2^2}\right]$$

$$= \|r^{(k)}-\hat{r}\|_2^2 \left[1 - \frac{1}{\|A\|_F^2}\frac{(r^{(k)}-\hat{r})'\sum_{j=1}^{n} a_j a_j'(r^{(k)}-\hat{r})}{\|r^{(k)}-\hat{r}\|_2^2}\right]$$

$$= \|r^{(k)}-\hat{r}\|_2^2 \left[1 - \frac{1}{\|A\|_F^2}\frac{(r^{(k)}-\hat{r})'AA'(r^{(k)}-\hat{r})}{\|r^{(k)}-\hat{r}\|_2^2}\right] \quad \text{(S.44)}$$

Since A is full-column rank, for any $x \in \mathbb{R}^m$, we have $\frac{x'AA'x}{\|x\|_2^2} \geq \frac{1}{\|A^\dagger\|_2^2}$ where $A^\dagger$ is the Moore—Penrose inverse of A. Therefore, (S.44) can be bounded above such that,

$$\mathbb{E}_{I(k+1)\big|\tilde{I}(k)}\left(\|r^{(k+1)}-\hat{r}\|_2^2\right) \leq \|r^{(k)}-\hat{r}\|_2^2 \left[1 - \frac{1}{\|A\|_F^2}\frac{1}{\|A^\dagger\|_2^2}\right]$$

$$\doteq (1-\kappa^{-2}(A))\|r^{(k)}-\hat{r}\|_2^2 \quad \text{(S.45)}$$

Notice that (S.45) holds for all $k \in \mathbb{N}$, which indicates that the square of the residual error shrinks at most by a constant factor $1-\kappa^{-2}(A)$ in each iteration. Therefore, given an initial residual $r^{(0)}$, expectation of the residual error over the product distribution $\tilde{I}(k)$ can be bounded by iteratively applying bound of conditional expectation of residual error shown in (S.45). In particular, by the definition of expectation over product distribution $\tilde{I}(k)$, we have

$$\mathbb{E}_{\tilde{I}(k)}\|r^{(k)}-\hat{r}\|_2^2 = \sum_{(s_1,\ldots,s_k)} f_{(I(1),\ldots,I(k)}(s_1,\ldots,s_k)\|r^{(k)}-\hat{r}\|_2^2. \quad \text{(S.46)}$$

Since $I(1), \dots, I(k)$ are independent, we have

$$f_{(I(1),\dots,I(k))(s_1,\dots,s_k)} = f_{(I(1),\dots,I(k-1))}(s_1, \dots, s_{k-1}) f_{I(k)}(s_k). \tag{S.47}$$

By the definition of conditional probability for independent random variables,

$$f_{I(k)\big|\tilde{I}(k)}(s_k \big| (s_1 \dots, s_{(k-1)})) = f_{I(k)}(s_k).$$

Therefore together with (S.47), (S.46) can be rewritten as

$$\begin{aligned}
\mathbb{E}_{\tilde{I}(k)} \|r^{(k)} - \hat{r}\|_2^2 &= \sum_{(s_1,\dots,s_{k-1})} \sum_{s_k} f_{(I(1),\dots,I(k-1))}(s_1, \dots, s_{k-1}) f_{I(k)\big|\tilde{I}(k-1)}(s_k) \|r^{(k)} - \hat{r}\|_2^2 \\
&= \sum_{(s_1,\dots,s_{k-1})} f_{(I(1),\dots,I(k-1))}(s_1, \dots, s_{k-1}) \sum_{s_k} f_{I(k)\big|\tilde{I}(k-1)}(s_k) \|r^{(k)} - \hat{r}\|_2^2 \\
&= \sum_{(s_1,\dots,s_{k-1})} f_{(I(1),\dots,I(k-1))}(s_1, \dots, s_{k-1}) \mathbb{E}_{I(k)\big|\tilde{I}(k-1)} \|r^{(k)} - \hat{r}\|_2^2, \qquad \text{(S.48)}
\end{aligned}$$

Substituting (S.45) into (S.48) yields

$$\begin{aligned}
\mathbb{E}_{\tilde{I}(k)} \|r^{(k)} - \hat{r}\|_2^2 &\le \sum_{(s_1,\dots,s_{k-1})} f_{(I(1),\dots,I(k-1))}(s_1, \dots, s_{k-1})(1 - \kappa^{-2}(A)) \|r^{(k-1)} - \hat{r}\|_2^2 \\
&= (1 - \kappa^{-2}(A)) \mathbb{E}_{\tilde{I}(k-1)} \|r^{(k-1)} - \hat{r}\|_2^2. \qquad \text{(S.49)}
\end{aligned}$$

By iteratively applying (S.49), one gets

$$\mathbb{E}_{\tilde{I}(k)} \|r^{(k)} - \hat{r}\|_2^2 \le (1 - \kappa^{-2}(A))^k \|r^{(0)} - \hat{r}\|_2^2,$$

which implies $r^{(k)}$ converges to $\hat{r}$ exponentially with respect to k in expectation.

Moreover, if A is of full column rank, then

$$\|r^{(k)} - \hat{r}\|_2^2 = \|A(x^{(k)} - \hat{x})\|_2^2 \ge \|A^\dagger\|_2^2 \|x^{(k)} - \hat{x}\|_2^2,$$

which implies $x^{(k)}$ converges to $\hat{x}$ exponentially with respect to k, since

$$\mathbb{E}_{\tilde{I}(k)} \|x^{(k)} - \hat{x}\|_2^2 \le \frac{1}{\|A^\dagger\|_2^2} \mathbb{E}_{\tilde{I}(k)} \|r^{(k)} - \hat{r}\|_2^2 \le \left(1 - \frac{1}{\kappa^2(A)}\right)^k \frac{\|r^{(0)} - \hat{r}\|_2^2}{\|A^\dagger\|_2^2}.$$

□

From hereon, we denote $\mathbb{E}_{\tilde{I}(k)}\|r^{(k)} - \hat{r}\|_2^2$ as $\mathbb{E}\|r^{(k)} - \hat{r}\|_2^2$ for brevity.

Theorem S.5. *Given the tolerance threshold, $\epsilon > 0$, and probability, $\delta \in (0, 1)$. Starting with an initial solution $x^{(0)}$ with the initial residual $r^{(0)} = b - Ax^{(0)}$, the total number of iterations required for the RRP algorithm to achieve $\|r^{(k)} - \hat{r}\|_2 \le \epsilon$ with a probability at least $1 - \delta$ is given by*

$$k > \log_{1-\kappa^{-2}(A)} \frac{\delta\epsilon^2}{\|r^{(0)}\|_2^2}. \tag{S.50}$$

Analogously, to achieve $\|x^{(k)} - \hat{x}\|_2 \le \epsilon$ with probability at least $1 - \delta$, the total number of iterations needed is

$$k > \log_{1-\kappa^{-2}(A)} \frac{\|A^\dagger\|_2^2 \, \delta\epsilon^2}{\|r^{(0)}\|_2^2}, \tag{S.51}$$

where $\kappa(A) = \|A\|_F \|A^{-1}\|_2$ is the modified condition number of A, $\|A\|_F$ is the Frobenius norm of A, and $A^\dagger$ is the Moore−Penrose inverse of A.

Proof. By the Markov's inequality, for any $\epsilon > 0$,

$$\begin{aligned} \mathbb{P}\left(\|r^{(k)} - \hat{r}\|_2 > \epsilon\right) &= \mathbb{P}\left(\|r^{(k)} - \hat{r}\|_2^2 > \epsilon^2\right) \\ &\le \frac{\mathbb{E}\|r^{(k)} - \hat{r}\|_2^2}{\epsilon^2}. \end{aligned} \tag{S.52}$$

By substituting the bound for $\mathbb{E}\|r^{(k)} - \hat{r}\|_2^2$ from (S.34) into (S.52), we get

$$\begin{aligned} \mathbb{P}\left(\|r^{(k)} - \hat{r}\|_2 > \epsilon\right) &\le \frac{\mathbb{E}\|r^{(k)} - \hat{r}\|_2^2}{\epsilon^2} \\ &\le \frac{(1 - \kappa^{-2}(A))^k \|r^{(0)} - \hat{r}\|_2^2}{\epsilon^2}. \end{aligned} \tag{S.53}$$

Notice that $r^{(0)}$ can be decomposed as $r^{(0)} = r_A + \hat{r}$ such that $r_A \in \mathcal{R}(A)$ and $\hat{r} \perp \mathcal{R}(A)$. Therefore we have $\|r^{(0)} - \hat{r}\|_2^2 = \|r_A\|_2^2 \le \|r^{(0)}\|_2^2$. Then the bound computed in (S.53) can be relaxed to get

$$\mathbb{P}\left(\|r^{(k)} - \hat{r}\|_2 > \epsilon\right) \le \frac{(1 - \kappa^{-2}(A))^k}{\epsilon^2} \|r^{(0)}\|_2^2.$$

In order to have $\mathbb{P}\left(\|r^{(k)} - \hat{r}\|_2 > \epsilon\right) < \delta$, we need $\frac{(1-\kappa^{-2}(A))^k}{\epsilon^2}\|r^{(0)}\|_2^2 < \delta$, which requires $k > \log_{(1-\kappa^{-2}(A))} \frac{\delta\epsilon^2}{\|r^{(0)}\|_2^2}$.

Analogously, in terms of the solution error, we have

$$\mathbb{P}\left(\|x^{(k)} - \hat{x}\|_2 > \epsilon\right) \leq \frac{\mathbb{E}\|x^{(k)} - \hat{x}\|_2^2}{\epsilon^2}. \tag{S.54}$$

When A is of full column rank, substituting the bound for $\mathbb{E}\|x^{(k)} - \hat{x}\|_2^2$ from (S.35) into (S.54) yields

$$\mathbb{P}\left(\|x^{(k)} - \hat{x}\|_2 > \epsilon\right) \leq \frac{\mathbb{E}\|x^{(k)} - \hat{x}\|_2^2}{\epsilon^2} \leq \left(1 - \kappa^{-2}(A)\right)^k \frac{\|r^{(0)} - \hat{r}\|_2^2}{\epsilon^2\|A^\dagger\|_2^2}.$$

In order to have $\mathbb{P}\left(\|x^{(k)} - \hat{x}\|_2 > \epsilon\right) < \delta$, we need $k > \log_{(1-\kappa^{-2}(A))} \frac{\|A^\dagger\|_2^2\delta\epsilon^2}{\|r^{(0)}\|_2^2}$. $\square$

S.4 Discussions on RRP

S.4.1 Incremental problems

Let $(\hat{x}_1, \hat{r}_1)$ be the solution-residual pair of $A_1x_1 = b_1$. We denote the expanded system due to additional rows or columns as $Ax = b$.

When additional rows A_2 are added to A_1, as modeled in (S.2), by Theorem S.5, starting with an initial solution-residual pair $(0, b_1)$ using the RRP algorithm for this expanded system, the total number of iterations required to reach the condition $\|r^{(k)} - \hat{r}\|_2 < \epsilon$ with a probability greater than or equal to $1 - \delta$ is $k > \log_{1-\kappa^{-2}(A)} \frac{\delta\epsilon^2}{\|b_1\|_2^2+\|b_2\|_2^2} \doteq k_1$. However, if this system is solved with the initialization, $x^{(0)} = \hat{x}_1$ and $r^{(0)} = \hat{r} = \begin{bmatrix} \hat{r}_1 \\ b_2 - A_2\hat{x}_1 \end{bmatrix}$, which utilizes the solution to $A_1x_1 = b_1$, the total number of iterations required to achieve the same accuracy is $\log_{1-\kappa^{-2}(A)} \frac{\delta\epsilon^2}{\|\hat{r}_1\|_2^2+\|b_2-A_2\hat{x}_1\|_2^2} \doteq k_2$. Note that $\hat{r}_1 \leq \|b_1\|_2$ and $\|b_2 - A_2\hat{x}_1\|_2 \leq \|b_2\|_2$ always hold. Hence $k_2 < k_1$.

Analogously, when more columns A_2 are added as presented in (S.4), and if the expanded system is solved with the solution-residual pair $(0, b_1 + b_2)$, the total number of iterations re-

quired to have $\|r^{(k)} - \hat{r}\|_2 < \epsilon$ with a probability of at least $1 - \delta$ is given by $k > k_3$, where $k_3 \doteq \log_{1-\kappa^{-2}(A)} \frac{\delta\epsilon^2}{\|b_1+b_2\|_2^2}$. On the other hand, if the expanded system is solved with an initial solution $\begin{bmatrix}\hat{x}_1 \\ 0\end{bmatrix}$ and the initial residual $r = \hat{r}_1 + b_2$, the total number of iterations required to achieve the same accuracy is given by $k > \log_{1-\kappa^{-2}(A)} \frac{\delta\epsilon^2}{\|\hat{r}_1+b_2\|_2^2} \doteq k_4$. In general $b_2 = 0$ and $\|\hat{r}_1\|_2 \leq \|b_1\|_2$ holds. Hence $k_4 < k_3$.

S.4.2 Decremental problems

Consider the decremental problem modeled as in (S.3), where a block of rows, $A_2 \in \mathbb{R}^{d\times n}$ is deleted. Suppose the least squares solution and the minimum residual of $\begin{bmatrix}A_1 \\ A_2\end{bmatrix} x = \begin{bmatrix}b_1 \\ b_2\end{bmatrix}$ are known as $\hat{x}$ and $\hat{r}$. Let r_1 be the residual obtained from $\hat{r}$ deleting last d terms. Then, if $A_1x = b_1$ is solved using RRP with an initial solution-residual pair $(0, b_1)$, the total iterations required is given as $k_5 = \log_{1-\kappa^{-2}(A_1)} \frac{\delta\epsilon^2}{\|b_1\|_2^2}$. In contrast, if the initialization is $x^{(0)} = \hat{x}, r^{(0)} = r_1$, number of iterations required (to solve the new LIP with the desired accuracy) becomes $k_6 = \log_{1-\kappa^{-2}(A_1)} \frac{\delta\epsilon^2}{\|r_1\|_2^2}$. Note that $\|r_1\|_2 \leq \|b_1\|_2$ always holds. Hence $k_5 \leq k_6$.

Similarly, if a block of columns, say $A_2 \in \mathbb{R}^{m\times d}$, is deleted, as modeled in (S.5). Then solving $A_1x = b_1$ as a new problem using the RRP starting from the solution-residual pair $(0, b_1)$ requires $k_7 = \log_{1-\kappa^{-2}(A_1)} \frac{\delta\epsilon^2}{\|b_1\|_2^2}$ iterations. On the other hand, by definition of minimum residual, $\hat{r} = b_1 - A\hat{x} = b_1 - A_1x_1 - A_2x_2$, where x_1 is the first $n - d$ terms of $\hat{x}$ and x_2 is the last d terms of $\hat{x}$. This is equivalent to

$$\hat{r} + A_2x_2 = b_1 - A_1x_1 \tag{S.55}$$

From (S.55), $A_1x = b_1$ can be solved using the RRP with initialization $x^{(0)} = x_1$ and $r^{(0)} = \hat{r} + A_2x_2$, which requires $k_8 = \log_{1-\kappa^{-2}(A_1)} \frac{\delta\epsilon^2}{\|\hat{r}+A_2x_2\|_2^2}$ iterations. If x_2 corresponds to an unimportant feature/node, then $x_2 \approx 0$, which implies $\|\hat{r} + A_2x_2\|_2 < \|b_1\|_2$. Hence $k_8 < k_7$.

S.4.3 Termination criterion

In practice, calculating the total number of iterations needed to compute the least-squares solution using (S.50) and (S.51) is not feasible due to the computational intractability of $\kappa(A)$. The expressions in (S.50) and (S.51) are useful for theoretical analysis only. Therefore, when implementing the RRP algorithm, a calculable termination condition is required.

An approximate slope of the residual can be used as an indicator to terminate the algorithm, i.e., given a tolerance $\epsilon > 0$, for some $h \in \mathbb{N}$, we can terminate the algorithm when the following condition is satisfied

$$\left\| r^{(k+h)} - r^{(k)} \right\|_2 < h\epsilon. \tag{S.56}$$

Alternatively, we could also define the termination condition in terms of the projection of the residual $r^{(k)}$ on the column space of the matrix A, i.e., given a tolerance $\epsilon > 0$, we can terminate the algorithm when the following condition is satisfied

$$\left\| A' r^{(k)} \right\|_2 < \epsilon. \tag{S.57}$$

As the minimum residual $\hat{r}$ is perpendicular to $\mathcal{R}(A)$, (S.57) is equivalent to

$$\| A'(r^{(k)} - \hat{r}) \|_2 < \epsilon. \tag{S.58}$$

If A has full column rank, then $\|A'(r^{(k)} - \hat{r})\|_2 \geq \|A^\dagger\|_2^{-1} \|r^{(k)} - \hat{r}\|_2$. Hence, the terminal condition in (S.57) yields

$$\|A^\dagger\|_2^{-1} \|(r^{(k)} - \hat{r})\|_2 < \epsilon \Rightarrow \|(r^{(k)} - \hat{r})\|_2 < \|A^\dagger\|_2 \epsilon, \tag{S.59}$$

which bounds the residual error.

S.5 Parallel RRP (PRRP) for LIPs

In addition to the developed PRP framework described in Section S.2, here we further propose an integrated parallel RRP (PRRP) algorithm. The PRRP algorithm is an iterative algorithm

for solving an LIP as in (S.1), which employs the RRP algorithm within the PRP framework to solve the residual problems defined in Section S.2. The pseudo code for the PRRP algorithm is shown in Algorithm S.3.

Algorithm S.3 Parallel randomized residual projection (PRRP)

```
function PRRP(A, b, p)
    Initialize: A = [A_1, ..., A_p], A_i ∈ R^{m×d_i}, R̂_i^{(0)} = (1/p) b, X̂_i^{(0)} = 0, i = 1, ..., p.
                w_1, ..., w_p such that 0 < w_i < 1 for i = 1, ..., p and Σ_{i=1}^p w_i = 1.
    for k ← 1, 2, ... do:
        R_i = w_i Σ_{i=1}^p R̂_i
        parfor i = 1, ..., p do
            δX̂_i, R̂_i = RRP(A_i, 0_{d_i}, R_i)
        end parfor
        X̂_i = X̂_i + δX̂_i
    end for
    return x = [X̂_1; ...; X̂_p], r = Σ_{i=1}^p R̂_i
end function
```

The convergence property and the rate of convergence of the PRRP algorithm follow directly from Theorem S.1. The following Lemma is used in analyzing the conditions for partitioning matrix A in the PRRP algorithm to achieve acceleration (due to the parallel computational architecture) for solving LIPs.

Lemma S.3. *Suppose $A_1 \in \mathbb{R}^{m\times n}$ has full column-rank and $w \in \mathbb{R}^m$. If the augmented matrix $A_2 = [A_1\ w]$ has full column-rank, then $\sigma_{min}(A_1) \geq \sigma_{min}(A_2)$, where $\sigma_{min}(A)$ is the smallest non-zero singular value of A.*

Proof. We denote the eigenvalues of $A_2'A_2$ as $\beta_1 \geq \cdots \geq \beta_n$, and the eigenvalues of $A_1'A_1$ as $\gamma_1 \geq \cdots \geq \gamma_{n-1}$. Since it holds that

$$A_2'A_2 = \begin{bmatrix} A_1'A_1 & A_1'w \\ w'A_1 & w'w \end{bmatrix},$$

we have $A_1'A_1$ is a principle submatrix of $A_2'A_2$. Then, by Cauchy interlacing theorem, we have

$$\beta_1 \geq \gamma_1 \geq \beta_2 \geq \cdots \geq \gamma_{n-1} \geq \beta_n$$

which implies $\sigma_{min}(A_1) = \sqrt{\gamma_{n-1}} \geq \sqrt{\beta_n} = \sigma_{min}(A_2)$. □

Lemma S.4. *Suppose A has full column-rank. If A is partitioned into column blocks, i.e. $A = [A_1, \ldots, A_p]$, then $\kappa(A) \geq \min_{i\in\{1,\ldots,p\}} \kappa(A_i)$.*

Proof. Note that for all $i \in \{1, \ldots, p\}$, we have $\|A_i\|_F \leq \|A\|_F$. From Lemma S.3, we have

$$\frac{\sigma^2_{min}(A_i)}{\|A_i\|_F} \geq \frac{\sigma^2_{min}(A)}{\|A\|_F} \Rightarrow \frac{1}{||A_i^{\dagger}||_2\|A_i\|_F} \geq \frac{1}{\|A^{\dagger}\|_2\|A\|_F} \Rightarrow \min_{i\in\{1,\ldots,p\}} \kappa(A_i) \leq \kappa(A).$$

□

Theorem S.6 (Accelaration by PRRP). *Given the tolerance threshold, $\epsilon > 0$, and probability, $\delta \in (0, 1)$. Starting with an initial residual $\hat{R}^{(k)}$, the computational time needed to achieve $\|\hat{R}^{(k)} - \hat{R}^{(k+1)}\|_2 \leq \epsilon$ with a probability at least $1 - \delta$ for $k = 0, 1, \ldots$, using the PRRP (Algorithm S.3) is less than the time taken by the RRP (Algorithm S.2) provided that the column partition of A (in Algorithm S.3) satisfies*

$$\max_i \big\|w_iA_i(A_i'A_i)^{-1}A_i'\hat{R}^{(k)}\big\|_2 \leq \big\|\sum_{i=1}^{p} w_iA_i(A_i'A_i)^{-1}A_i'\hat{R}^{(k)}\big\|_2.$$

Proof. From Theorem S.5, the computational time needed to complete one stage in the PRRP algorithm can be computed as the time required to complete k^* iterations, where

$$k^* > \max_{i\in\{1,\ldots,p\}} \log_{1-\kappa^{-2}(A_i)} \frac{\delta\epsilon^2}{\|w_i\hat{R}^{(k)} - \hat{R}_i^{(k+1)}\|_2^2} = \log_{1-\kappa^{-2}(A_{i^*})} \frac{\delta\epsilon^2}{\|w_i\hat{R}^{(k)} - \hat{R}_{i^*}^{(k+1)}\|_2^2}, \quad \text{(S.60)}$$

with $i^* = \underset{i}{\operatorname{argmax}} \log_{1-\kappa^{-2}(A_i)} \frac{\delta\epsilon^2}{\|w_i\hat{R}^{(k)} - \hat{R}_i^{(k+1)}\|_2^2}$. Since $\kappa(A_i) \leq \kappa(A)$ (from Lemma S.4), we have $1 - \kappa^{-2}(A_i) \leq 1 - \kappa^{-2}(A)$ for $i = 1, \ldots, p$. Therefore, from (S.60), we have

$$\begin{aligned}\log_{1-\kappa^{-2}(A_{i^*})} \frac{\delta\epsilon^2}{\|w_i\hat{R}^{(k)} - \hat{R}_{i^*}^{(k+1)}\|_2^2} &\leq \log_{1-\kappa^{-2}(A)} \frac{\delta\epsilon^2}{\|w_i\hat{R}^{(k)} - \hat{R}_{i^*}^{(k+1)}\|_2^2} \\ &\leq \log_{1-\kappa^{-2}(A)} \frac{\delta\epsilon^2}{\max_i \|w_i\hat{R}^{(k)} - \hat{R}_i^{(k+1)}\|_2^2}.\end{aligned}$$

On the other hand, with the RRP algorithm, the number of iterations required to reduce the initial residual $\hat{R}^{(k)}$ until the residual becomes $\hat{R}_1^{(k+1)} + \ldots + \hat{R}_p^{(k+1)}$ is lower bounded by $\log_{1-\kappa^{-2}(A)} \frac{\delta\epsilon^2}{\|\hat{R}^{(k)} - \sum_i \hat{R}_i^{(k+1)}\|_2^2}$.

If given a partition of $A = \left[A_1, \ldots, A_p\right]$, it holds that

$$w_i \hat{R}^{(k)} - \hat{R}_i^{(k+1)} = A_i \left(A_i^T A_i\right)^{-1} A_i^T w_i \hat{R}^{(k)},$$

since $w_i \hat{R}^{(k)} - \hat{R}_i^{(k+1)}$ is the projection of $\omega_i \hat{R}^{(k)}$ onto $\mathcal{R}(A_i)$.

Therefore, when $\max_i \|w_i \hat{R}^{(k)} - \hat{R}_i^{(k+1)}\|_2^2 \leq \|\hat{R}^{(k)} - \sum_i \hat{R}_i^{(k+1)}\|_2$, we have

$$\max_i \|w_i A_i \left(A_i' A_i\right)^{-1} A_i' \hat{R}^{(k)}\|_2 \leq \|\sum_i w_i A_i (A_i' A_i)^{-1} A_i' \hat{R}^{(k)}\|_2. \tag{S.61}$$

If $\max_i \|\omega_i \hat{R}^{(k)} - \hat{R}_i^{(k+1)}\|_2^2 \leq \|\hat{R}^{(k)} - \sum_i \hat{R}_i^{(k+1)}\|_2$ holds, then

$$\begin{aligned} \max_{i \in \{1,\ldots,p\}} \log_{1-\kappa^{-2}(A_i)} \frac{\delta\epsilon^2}{\|w_i \hat{R}^{(k)} - \hat{R}_i^{(k+1)}\|_2^2} &\leq \log_{1-\kappa^{-2}(A)} \frac{\delta\epsilon^2}{\max_i \|w_i \hat{R}^{(k)} - \hat{R}_i^{(k+1)}\|_2^2} \\ &\leq \log_{1-\kappa^{-2}(A)} \frac{\delta\epsilon^2}{\|\hat{R}^{(k)} - \sum_i \hat{R}_i^{(k+1)}\|_2^2}, \end{aligned}$$

which implies that the PRRP algorithm expedites the residual projection. □

S.6 Experiments

In this section, we provide several numerical experiments to demonstrate the efficacy of the proposed algorithm to solve a large-scale linear inverse problem as in (S.1). All the numerical experiments were implemented on a single workstation with Xeon Gold 6144 3.5GHz, 192GB memory.

S.6.1 Example 1: Performance analysis of the RRP and the PRP

We generated A as a random matrix of dimension $100,000 \times 50,000$ (≈ 40 GB memory storage in MATLAB). Each element of A was random under a uniform distribution on $[0, 1]$. A sparse

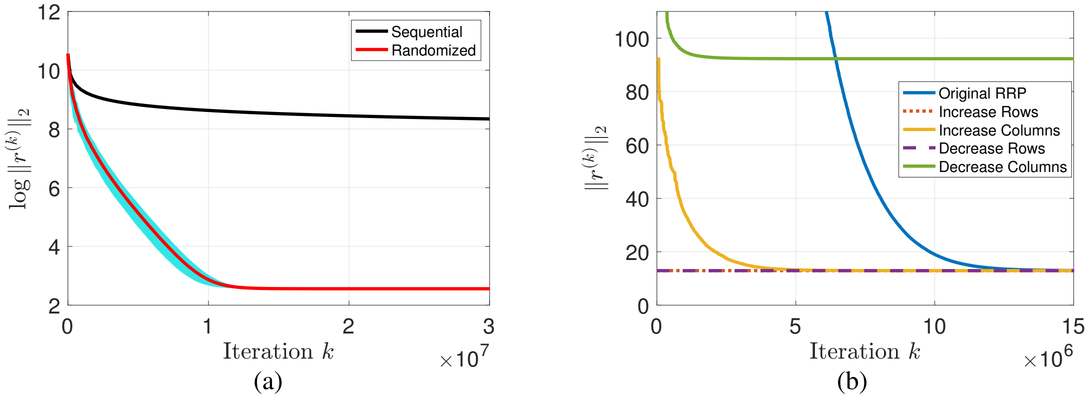

Figure S.1: (a) Comparison between the RRP algorithm and the sequential residual projection algorithm (columns in each iteration are selected sequentially) for solving (S.1) with the size of A fixed at $100,000 \times 50,000$. The solid red line is the mean residual calculated over 10 simulation experiments with the RRP algorithm, while the blue fill is the deviation from the mean error. (b) Demonstration of scalability of the RRP algorithm: the solid blue line shows the convergence of the residual while solving (S.1) with the size of A fixed at $100,000 \times 50,000$; the dashed purple line and solid green line indicate the convergence of the norm of the residual with the RRP algorithm for adaptively solving the decremental problems as in (S.3) and (S.5), when the last 100 rows and columns of A were deleted, respectively; the dotted red line and solid yellow line indicate the convergence of the norm of the residual with the RRP algorithm for adaptively solving the incremental problems as in (S.2) and (S.4), when the 100 rows and columns deleted in the decremental problems were added back, correspondingly.

vector z of dimension $50,000 \times 1$ was then generated which consisted of 99% of elements as 0's, 1's as the remaining elements. The vector of observations, b was generated by $b = Az + \omega$, where ω was selected as a $100,000 \times 1$ random vector with elements uniformly distributed in $[0, 0.2]$. When running the RRP algorithm, the termination criterion was chosen as (S.57) with $\epsilon = 2.236 \times 10^{-4}$ and it was checked every $500,000$ iterations.

In Figure S.1(a), we demonstrate the exponential convergence of the RRP algorithm described in Theorem S.4, where the logarithmic scale of the norm of residuals following randomized and sequential- residual projection iterations are plotted. We observe that a random choice of the column index at each iteration significantly improves the rate of convergence compared to the sequential choice.

In Figure S.1(b), we demonstrate the scalability of the RRP algorithm to solve the incremental/decremental problems as in (S.2)-(S.5) by *adaptively* updating the solutions as the size

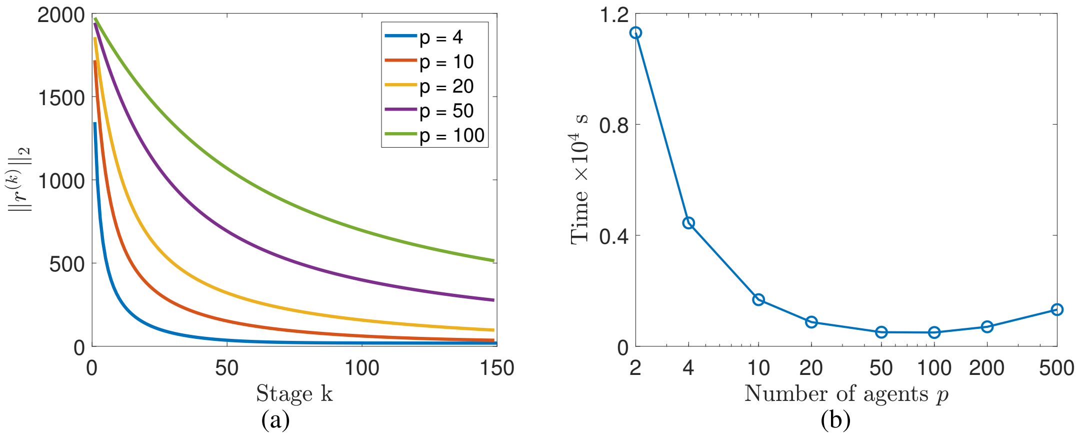

Figure S.2: (a) Residual convergence for the PRP under various numbers of agents, where the matrix A of size $100,000 \times 50,000$ was partitioned into p columns blocks of size $100,000 \times 50,000/p$ in each case. (b) Convergence time (in s) when the total residual becomes smaller than 0.5% of the initial residual using the PRP algorithm with various number of partitions p.

of the matrix A changes. In the incremental cases, the size of the matrix A was increased from $100,000 \times 49,900$, $99,900 \times 50,000$, to $100,000 \times 50,000$, corresponding row increment and column increment, respectively. Similarly in the decremental cases, the size of A was decreased from $100,000 \times 50,000$, to $100,000 \times 49,900$ and $99,900 \times 50,000$. It can be observed that for solving the problems arising from a change in the size of the matrix A, updating the existing solution, residual *adaptively* is computationally more efficient. The time required to obtain a solution, the number of iterations needed to terminate the RRP algorithm, and the solution error are recorded in Table S.1.

Case	Running time (s)	Iterations	$\|x^{(k)} - \hat{x}\|_\infty$
RRP	7,368	4.2×10^7	3.7×10^{-2}
Row increment	3,377	1.9×10^7	1.9×10^{-3}
Column increment	5,741	3.3×10^7	3.7×10^{-3}
Row decrement	3,702	2.1×10^7	3.7×10^{-2}
Column decrement	5,582	3.2×10^7	2.9×10^{-2}

Table S.1: Running time, number of iterations needed to terminate the RRP algorithm, and the solution error for the incremental/decremental problems.

We analyzed the PRP algorithm for varying numbers of agents, and the results are presented in Figure S.2(a). We first fixed the size of A to be $100,000 \times 50,000$. Then the matrix A was decomposed into p column blocks, i.e. $A = [A_1, \ldots, A_p]$, where each column block was of dimension $100,000 \times 50,000/p$. It is observed that as the number of partitions p increases, the residual convergence is slower with respect to the number of stages. However, as the number of partitions is increased, the size of each sub-problem is reduced. Therefore, to analyze the effect of the partition size on the convergence of the algorithm, we considered the time required to reduce the residual to 0.5% of the initial residual and the results are depicted in Figure S.2(b) for various partition sizes.

Specifically, we fixed the size of the matrix A as $100,000 \times 50,000$ while varying p, and each sub-problem was solved by directly computing the pseudo-inverse of A_i. The time taken to reduce the initial total residual until it is smaller than 0.5% of the initial residual using the parallel algorithm is recorded for different partition sizes in Figure S.2(b). It is discerned that as the number of partitions p increases, the time for convergence of the residual is initially reduced since the size of each sub-problem is reduced, then the acceleration seen in the convergence of the residual due to more partitions becomes insignificant, even negative when p is greater than 100 (for the example considered) as shown in Figure S.2(a). This follows from Theorem S.4, wherein the rate of convergence for the parallel algorithm is determined by the matrix $B \doteq I - \frac{1}{p}\sum_{i=1}^{p} A_i(A_i'A_i)^{-1}A_i'$, and it is observed in the numerical experiment that as the number of partitions increase, $\max_i \lambda_i$ in (S.21) becomes close to 1, which decelerates the convergence of the residual and magnifies the round-off error. Therefore, choosing an optimal number of partitions for the parallel algorithm is subject to a compromise among the computation time per stage, the number of stages required, and the numerical round-off error.

S.6.2 Example 2: Network inference

Network inference is a critical task encountered in various disciplines such as biology, social science, finance, etc. In this example, we provide numerical experiments using the PRP to solve linear inverse problems encountered in the network inference application involving dynamical systems.

We consider a network inference problem for a large-scale interconnected system composed of dynamical subsystems that interact with each other. These interactions among the subsystems can be represented with a network graph, wherein each node corresponds to a subsystem, and each edge in the graph represents the interactions/coupling between the subsystems. The objective considered in the network inference problem is to recover the topology of the network, and to find the significant edges (connections/couplings) using the time-series observations of each subsystem corresponding to each node in the network graph.

For example, consider an interconnected system with N dynamical subsystems each of which are represented as

$$\dot{x}_j(t) = g_j(x_j(t)) + \sum_{i=1}^{N} h_{ji}(x_i(t), x_j(t)), \quad j = 1, 2, .., n, \tag{S.62}$$

where $x_j \in \mathbb{R}$ represents the state of jth the subsystem, g_j represents the unknown internal dynamics, and h_{ji} represents the unknown nonlinear map modelling the interconnection between subsystems j and i. By approximating g_j and h_{ji} with linear combination of truncated orthonormal basis functions l and s respectively, one has

$$\dot{x}_j(t) \approx \sum_{p=0}^{u} r_j^{(p)} l^{(p)}(x_j(t)) + \sum_{\substack{i=1 \\ i \neq j}}^{N} \sum_{q=0}^{v} k_{ji}^{(q)} s^{(q)}(x_i(t), x_j(t)), \qquad j = 1, 2, .., n, \tag{S.63}$$

where $u, v \in \mathbb{N}$, $r_j^{(p)}, k_{ji}^{(q)}$ for $p = 0, \ldots, u$, and $q = 0, \ldots, v$ are unknown coefficients. Given the state observations from t_0 to t_m, i.e. $x_1(t_i), \ldots, x_N(t_i), i = 0, \ldots, m$, from (S.63), we have

a system of linear equations given by

$$\begin{bmatrix} \frac{x_j(t_1)-x_j(t_0)}{t_1-t_0} \\ \vdots \\ \frac{x_j(t_N)-x_j(t_{N-1})}{t_N-t_{N-1}} \end{bmatrix} = \begin{bmatrix} L(x_j(t_1)) & S(x_j(t_1)) \\ L(x_j(t_2)) & S(x_j(t_2)) \\ \vdots & \vdots \\ L(x_j(t_m)) & S(x_j(t_m)) \end{bmatrix} \begin{bmatrix} R_j \\ K_j \end{bmatrix}. \tag{S.64}$$

where $L(x_j(t_k)) = \begin{bmatrix} l^{(1)}(x_j(t_k)) \ldots l^{(u)}(x_j(t_k)) \end{bmatrix}$, $S(x_j(t_k)) = \begin{bmatrix} s^{(1)}(x_1(t_k),x_j(t_k) \ldots s^{(p)}(x_N(t_k),x_j(t_k)) \end{bmatrix}$, $R_j = \begin{bmatrix} r_j^{(1)} \\ \vdots \\ r_j^{(u)} \end{bmatrix}$ and $K_j = \begin{bmatrix} k_{1j}^{(1)} \\ \vdots \\ k_{Nj}^{(p)} \end{bmatrix}$.

Solving (S.64) for $j = 1, \ldots, N$ returns all unknown parameters $r_j^{(p)}, k_{ji}^{(q)}$ in (S.63). From a network point of view, $k_{ji}^{(q)}, q = 1 \ldots, v$ denotes how x_j is coupled with x_i. In particular, if $k_{ji}^{(q)}$ is large, then x_i and x_j are considered to be strongly coupled. It can be observed from (S.64) that the problem of network inference requires solving a suite of large-scale linear inverse problems (for $j = 1, \ldots, N$).

For the experiments, we considered a system of coupled oscillators, and the oscillator dynamics are represented using Kuramoto model (*32*). The dynamics of the ith oscillator in the network are given by

$$\dot{x}_j = \omega_j + \sum_{i=1}^{N} k_{ji} \sin(x_j - x_i). \tag{S.65}$$

It can be observed that (S.65) is a simplified case of (S.62), where $g_j(x_j) = \omega_j$ and $h_{ji}(x_i, x_j) = k_{ji} \sin(x_j - x_i)$.

To assign the values for k_{ji} in (S.65), we first generated a small-world network based on Watts-Strogatz model (*31*). The Watts-Strogatz model is characterized by the total number of nodes (N), the mean-degree (K), and the rewiring probability (β) such that

(a) each node i is connected with K nearest neighbors, i.e. $x_{i-K/2}, \ldots, x_{i+K/2}$, and

(b) each connection generated in step (a), with probability β, is replaced with another connection uniformly chosen from all possibilities that avoid self-loop and link duplications.

Let the adjacency matrix of the network be denoted by H. The total number of nodes were fixed as 300. We considered two cases corresponding to different values of β and K resulting in either a dense or a sparse network.

To recover the topology of the network, denote the solution of (S.64) as $\hat{X}$ to infer the coupling gains k_{ji}. Then, the matrix $\mathcal{K} = 0.5(\hat{X} + \hat{X}')$ was computed to infer the topology of the network. Specifically the topology of the network was recovered by constructing an adjacency matrix $\hat{H}$ such that

- $\hat{H}(i,j) = 1$ if $\mathcal{K}(i,j) > \hat{\mathcal{K}} + \hat{\mathcal{K}}_\delta$;
- $\hat{H}(i,j) = 0$ otherwise,

where $\hat{\mathcal{K}}$ is the average of the entries of the matrix $\mathcal{K}$ and $\hat{\mathcal{K}}_\delta$ is the standard deviation.

Dense network: The Watts-Strogatz network model was used with $N = 300, K = 20, \beta = 0.3$ to generate the topology $H(j,i)$ of a Kuramoto oscillator network. The coupling strength was selected as follows: if $H(i,j) = 0$, then $k_{ji} = 0.2$, and if $H(i,j) = 1$, the value of k_{ji} was uniformly distributed in $[0.4, 0.6]$. Hence, the above network is a densely connected network. Each oscillator in the network was defined using (S.65) with ω_j uniformly distributed in $[5, 10]$.

The estimates of the coupling gains were obtained by using the PRP algorithm to solve the linear inverse problems and the estimates are recorded in Figure S.3. The algorithm was run for 450 stages and it can be observed that the coupling gains are partially recovered. Recovering each of the coupling gains warrants stringent requirements in terms of data samples required, and the computation time.

Alternatively, the topology of the network can be inferred by computing an estimate of the adjacency matrix, $\hat{H}$. This provides the information regarding the network graph that maps the interconnections and the strength of the interconnections between the oscillators and this

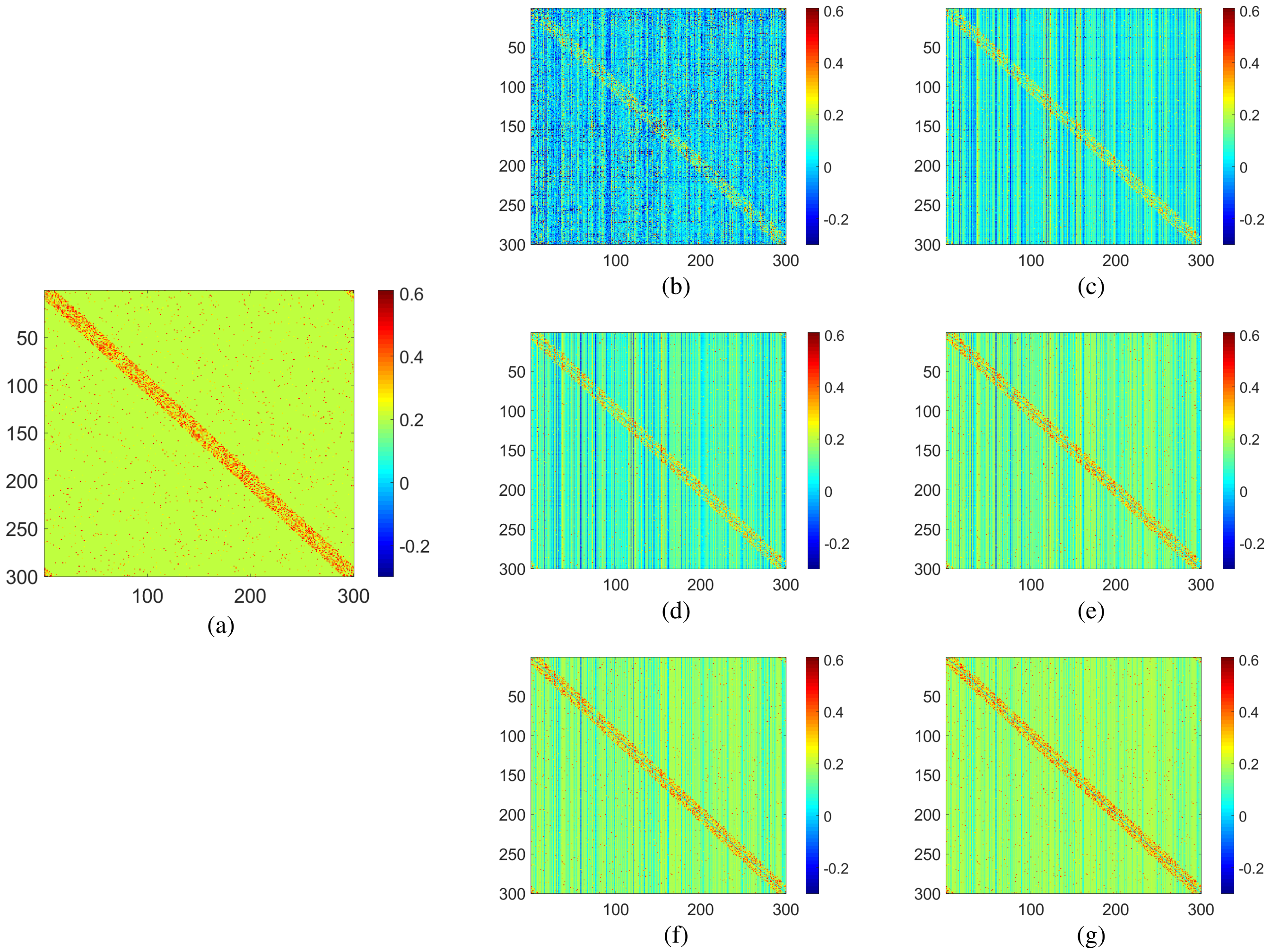

Figure S.3: (a) Original coupling strengths of dense Kuramoto oscillator network. (b)-(g) Estimated coupling strengths using the PRP algorithm after 10, 50, 100, 200, 300, and 450 stages.

requires less stringent data compared to the estimation of the coupling gains. The estimated topology of the network recovered using the PRP algorithm is recorded in Figure S.4.

The histogram of the degree distribution of the recovered and original network graph shown in Figure S.5(a) and the graph representation in Figure S.5(b), reveals that the strong couplings are recovered accurately. In a large network inference problem, depending on the required objective, for example, inference of the topology, inferring only the strong couplings, or estimating

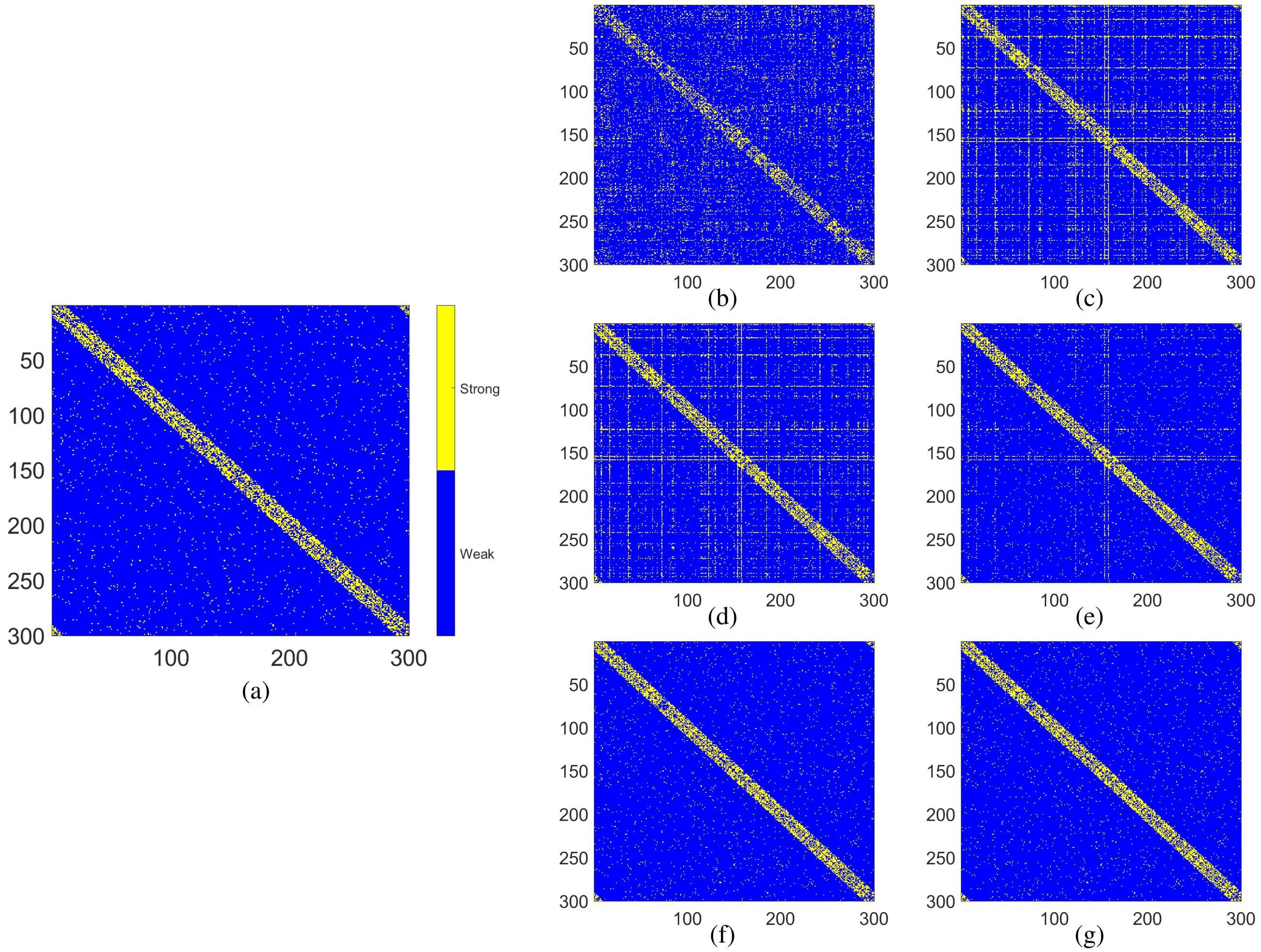

Figure S.4: (a) Original topology of dense Kuramoto oscillator network indicating if the connections are either strong or weak. Each point indicates whether two nodes are strongly or weakly coupled. (b)-(g) Network topology recovered using the PRP algorithm after 10, 50, 100, 200, 300, 450 stages.

all the coupling gains, the PRP algorithm can be terminated by providing the desired tolerance ϵ in (S.57).

Sparse Network: In this case, the Watts-Strogatz network model with $N = 100, K = 10, \beta = 0.3$ was used to generate the topology $H(j, i)$ of a Kuramoto oscillator network. The coupling strength was defined as follows: if $H(j, i) = 0$, then $k_{ji} = 0$, and if $H(j, i) = 1$, the value of k_{ji} was uniformly distributed in $[0.4, 0.6]$. Each oscillator in the network was defined

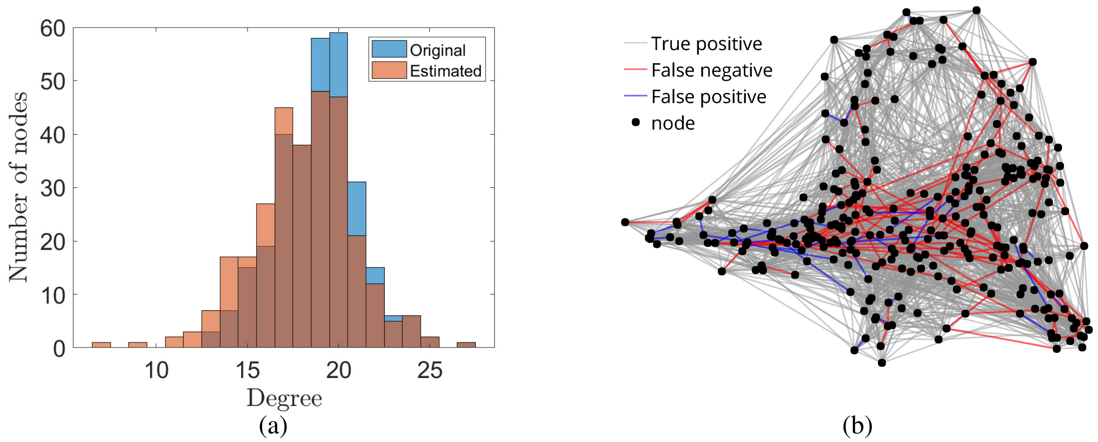

Figure S.5: (a) Degree histograms for the original and the estimated network after 450 stages for the dense Kuramoto oscillator network. Normalized Wasserstein distance of two histogram is 5.8×10^{-3}. (b) Topology recovered for the dense network after 450 stage; true positive indicates strong coupling recovered as strong one; false negative means strong coupling recovered as weak; false positive implies weak coupling recovered as strong one.

using (S.65) with ω_j uniformly distributed in $[5, 10]$.

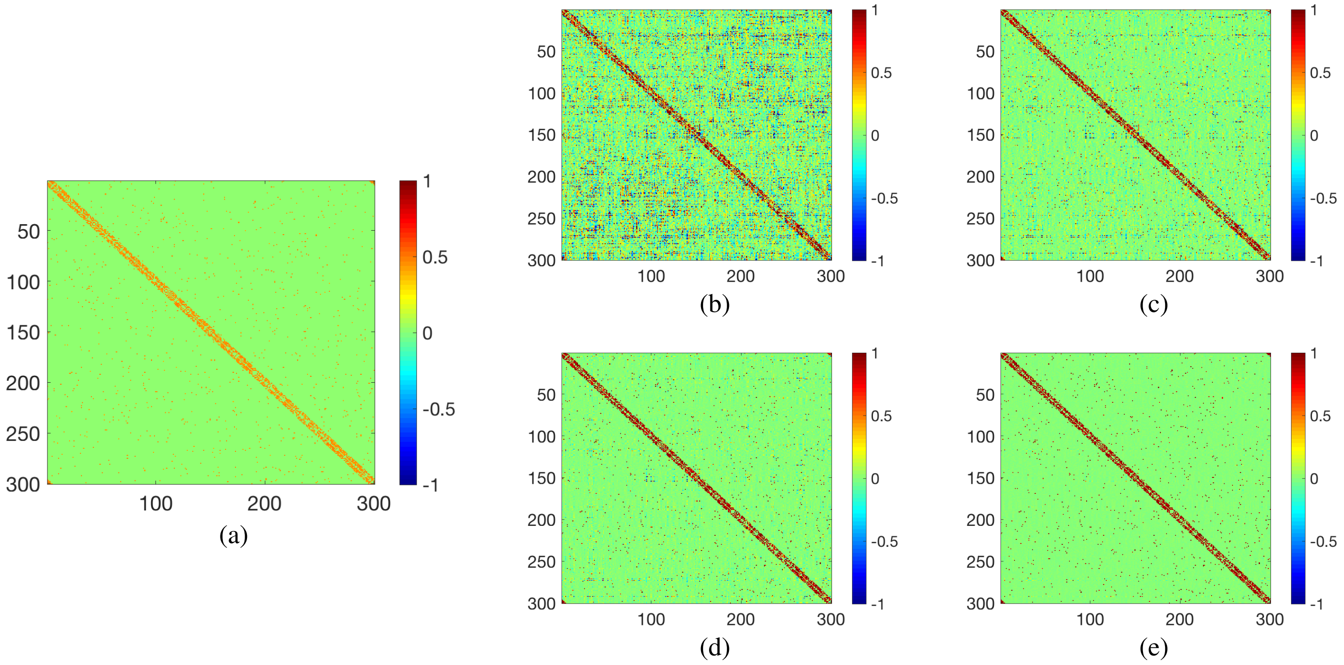

Figure S.6: (a) Original coupling strengths of a sparse Kuramoto oscillator network. (b)-(e) Coupling strengths estimated for the sparse network by using the PRP algorithm after 10, 30, 50, and 100 stages.

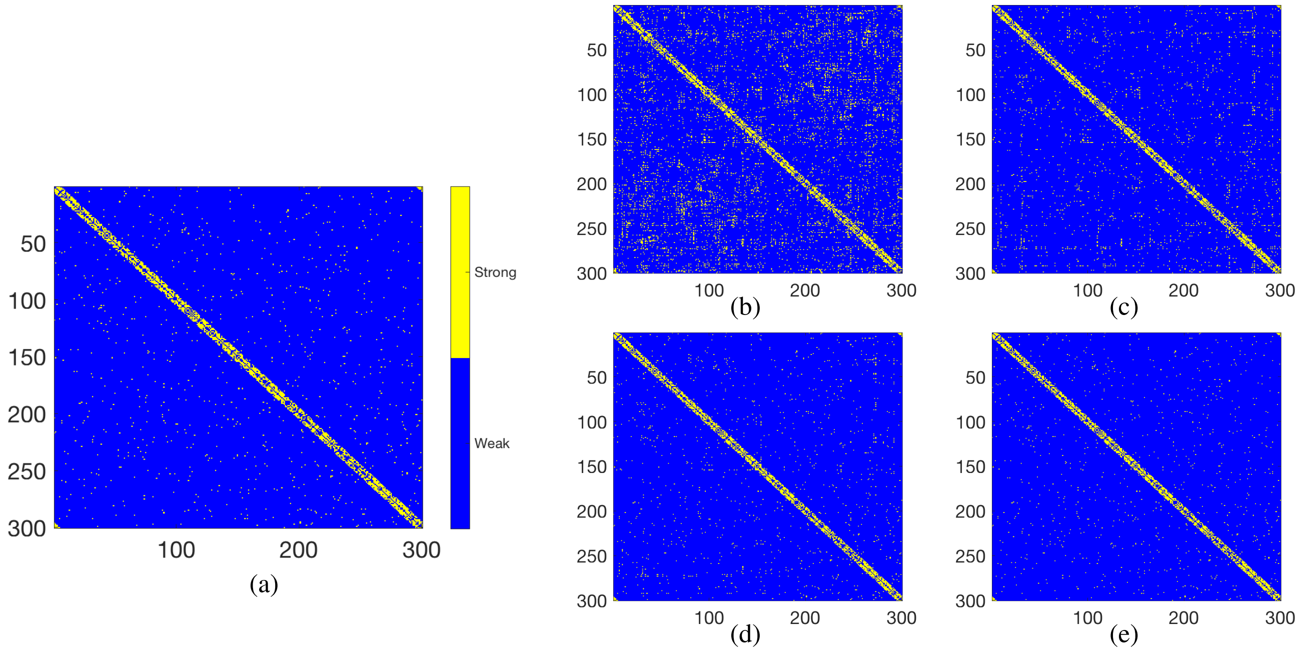

Figure S.7: (a) Original topology of a sparse Kuramoto oscillator network. (b)-(e) Coupling strengths estimated for the sparse network using the PRP algorithm after 10, 30, 50, and 100 stages.

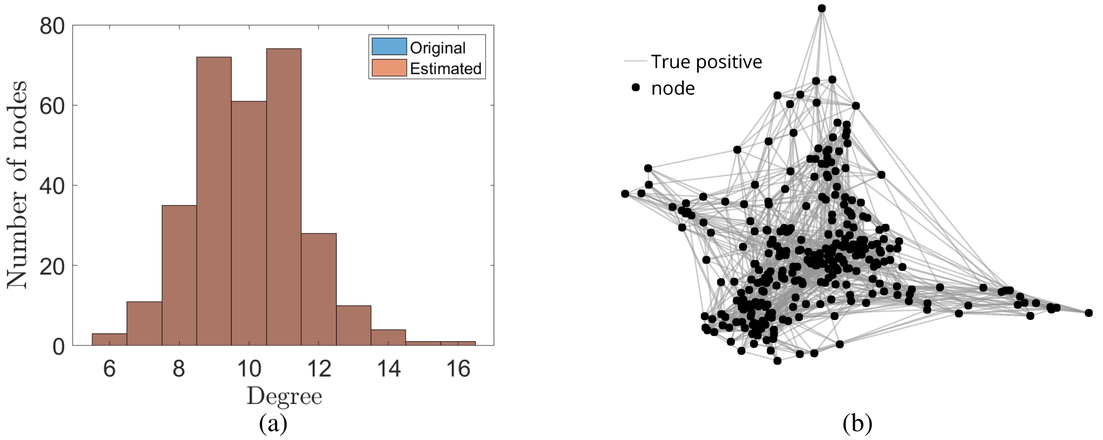

Figure S.8: (a) Degree histograms for the original and the estimated network after 100 stages for the sparse network. Normalized Wasserstein disntance of two histogram is 0, which indicates the network topology was perfectly recovered. (b) Topology recovered for sparse network after 100 stages.

The estimates of the coupling gains were obtained by using the PRP algorithm to solve the linear inverse problems are recorded in Figure S.6. The algorithm was run for 100 stages and it

can be observed that the coupling gains were recovered accurately. The estimated topology of the network recovered using the PRP algorithm is recorded in Figure S.7.

It can be observed from the degree histogram in Figure S.8(a) and graph representation in Figure S.8(b) that after 100 stages, the network topology was perfectly recovered when the associated LIPs were solved using the PRP.

Dense network		
Original \ Recovered	Strong	Weak
Strong	5352	320
Weak	80	84248

Sparse network		
Original \ Recovered	Strong	Weak
Strong	3000	0
Weak	0	87000

Table S.2: Topology recovered for the dense (after 450 stages) and the sparse network (after 100 stages) using the PRP algorithm.

Finally, the total edges in the graph recovered and the inference made regarding the strength of the interaction between two nodes for both the dense and the sparse network are tabulated (see Table S.2). It can be observed that the inference made on a sparse network was accurate while for the dense network, out of the 5672 strong connections, 5352 were correctly identified while the remaining 320 were inferred as weak connections, (94.3% accuracy) while the weak connections had better accuracy. As more data/observations are collected, since the PRP algorithm is flexible, the solution of the associated linear inverse problems can be incrementally and adaptively updated. This is applicable even when a new network node is introduced or an existing node is removed.

S.6.3 Example 3: Gravimetric survey

In geophysics, recovering Earth's gravitational field, Earth's magnetic field and seismic waves from earthquakes can all be formulated as linear inverse problems based on the observed data. We provide an example in which large masses in the subsurface can be detected using the measured gravitational field. We observe that detecting additional mass and re-computing mass

distribution in this case can be formulated as incremental/decremental problems as described in (S.2)-(S.5). To begin with, we provide a brief introduction on the problem setup.

Due to inhomogeneity of lithology in the subsurface of the Earth, the gravity measured on surface changes slightly across different locations. Such perturbations in the gravitational field can be used to infer the mineralogical composition underground (*33*). Suppose we have placed p gravimeters on the surface of the Earth which provides the acceleration due to gravity at p different locations which are denoted as $\vec{d}_1, \ldots, \vec{d}_p$. Assume that the locations of q objects in the subsurface are known a priori, while the mass of these objects are the unknown variables. From Newton's law of gravity, the gravity caused by q masses at location of ith gravimeter can be computed by

$$\vec{g}_i = \sum_{i}^{q} \frac{G m_j}{\|\vec{r}_{ij}\|^3} \vec{r}_{ij}, \tag{S.66}$$

where G is the universal gravitational constant; m_j is the jth unknown quantity of mass; $\vec{r}_{ij}$ is the distance from the ith gravimeter to the jth unknown mass. Applying (S.66) to all p gravimeters yields a linear equation in $m_j, j = 1, \ldots, q$, and hence, can be represented as

$$\begin{bmatrix} \vec{g}_1 \\ \vdots \\ \vec{g}_p \end{bmatrix} = \begin{bmatrix} \frac{G}{\|\vec{r}_{11}\|^3} \vec{r}_{11} & \cdots & \frac{G}{\|\vec{r}_{1q}\|^3} \vec{r}_{1q} \\ \vdots & & \vdots \\ \frac{G}{\|\vec{r}_{p1}\|^3} \vec{r}_{p1} & \cdots & \frac{G}{\|\vec{r}_{pq}\|^3} \vec{r}_{pq} \end{bmatrix} \begin{bmatrix} m_1 \\ \vdots \\ m_q \end{bmatrix} \tag{S.67}$$

In the problem stated as in (S.67), the locations of the objects in the subsurface are assumed to be known a priori, which is not practical in most geophysical applications. If (S.67) does not provide a satisfying solution, the prior assumption on number of objects, locations of objects needs to be modified, which results in incremental/decremental problems. Figure S.9(a) demonstrates an example of changing prior assumptions. Suppose an estimate of the mass of five objects inside the block is already obtained and if an additional object is detected, then, estimation of all six objects' mass can be quickly updated by an incremental problem; on the

other hand, if the object labeled by red cross is proven not to exist, then the estimation of the remaining four objects' mass can be updated by the decremental adaptation of the solution.

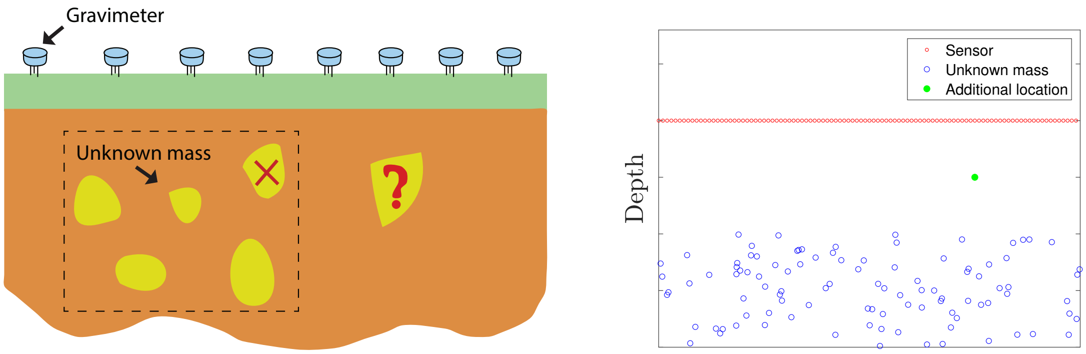

(a) (b)

Figure S.9: (a) Demonstration of estimating unknown masses in the subsurface. Suppose there are five masses in the dashed block as priori and if additional mass is detected, then estimating all six masses can be formulated as an incremental problem; while if one mass is proved to be non existent, estimating the remaining four masses can be formulated as a decremental problem. (b) Locations of gravimeters and unknown masses in the numerical experiments. Red circles indicate location of gravimeters, which are uniformly distributed on the surface; blue circles show where the unknown masses are in the subsurface; green dot represents the location where an additional mass is detected. For ease of exposition, only 100 gravimeters and 100 unknown masses are plotted in this figure.

We generated a 2-D example to show that the incremental problem can be expeditiously solved using the PRRP. Figure S.9(b) shows the locations of the gravimeters and the objects (only 100 gravimeters and objects are plotted here). For simplicity of calculation, we normalize G to 1. When applying the RRP algorithm, the termination criterion was chosen as in (S.57) with $\epsilon = 10^{-6}$. In our numerical experiments, we chose $p = 20,000$, $q = 10,000$ and the masses $m_1, \ldots, m_p$ were uniformly distributed from 0 to 1000; gravimeters were uniformly distributed on the horizontal level (depth equals to 0); depths of objects location were uniformly distributed from 1 to 2. Gravity measurements $g_1, \ldots, g_p$ was then computed using (S.66).

Given the gravity measurements, suppose the locations of the objects are already known, then solving linear inverse problem (S.67) to estimate their masses required 181.33 seconds. If an additional object's mass need to be estimated, indicated by a green circle, it took only 9.8036

seconds for the PRRP algorithm to provide a solution for the incremental problem by adaptively updating the existing solution.

References

32. Y. Kuramoto, *Chemical Oscillations, Waves, and Turbulence* (Courier Corporation, 2003).

33. T. H. Jordan, J. N. Franklin, Optimal solutions to a linear inverse problem in geophysics. *Proceedings of the National Academy of Sciences* **68**, 291–293 (1971).
